# Piloting a NGO-led signposting intervention to improve access to government welfare in Southern Morocco: a feasibility study

**DOI:** 10.1186/s12939-025-02605-0

**Published:** 2025-10-16

**Authors:** Fadi Baghdadi, Abdellah Soussi, Christopher Hands

**Affiliations:** 1 Faculty of Medical and Health Sciences, Institute of Life Science 2, Swansea University, Sketty, Swansea, SA2 8QA UK; 2 Fondation Amane pour la Protection de L’Enfance, DerbAqqa (À Coté d’hôtel Taroudant, N° 04 Derb ErragragaPlace El Alaouiine), Taroudant, Morocco; 3 Faculty of Medical and Health Sciences, University of Auckland, 85 Park Road, Grafton, Auckland, 1023 New Zealand

**Keywords:** Community-based participatory research, Community health planning, Community health services, Feasibility studies, Health equity, Health services accessibility, Morocco, Pilot projects, Public health systems research, Social welfare

## Abstract

**Background:**

Access to healthcare and social protection is a key determinant of health equity. In Morocco illiteracy, low awareness, and complex administrative processes contribute to poor enrolment in state welfare programmes, particularly in rural–urban migrant communities. In response, Morocco is transitioning to a consolidated social welfare model – *Registre Social Unifié* (RSU) – to promote more equitable access. We assessed the feasibility of a Non-Governmental-Organisation (NGO)-led signposting intervention to increase awareness of and access to government social welfare programmes in Southern Morocco.

**Methods:**

We used a participatory approach to design and deliver the study in two communes on the outskirts of Agadir, Southern Morocco. We conducted 18 social programme workshops with 1,087 parents, providing information on government welfare programmes. The intervention involved a four-stage process: community sensitisation, individual intake, referral to relevant programmes, and follow-up. We collected anonymised service data and qualitative feedback to evaluate demand, enrolment outcomes, and barriers to access.

**Results:**

Seventy percent (*n* = 785) of participants requested enrolment support. We successfully connected 67% to their desired programme, with the highest completion rates for RAMed (81%) and Tayssir (66%). Barriers included lack of civil registration, inconsistent documentation requirements, and limited coordination between agencies. Frontline staff also identified the absence of a formal social work framework as a barrier to sustainable implementation.

**Conclusions:**

This feasibility study indicates that an NGO-led signposting model may support improved access to social welfare in vulnerable Moroccan communities. As Morocco implements the RSU, aiming for full coverage by 2030, integrating an NGO-led outreach model and strengthening social work infrastructure could address enrolment barriers. Future research should examine the long-term impact and scalability of community-based interventions to advance health equity in Morocco.

**Supplementary Information:**

The online version contains supplementary material available at 10.1186/s12939-025-02605-0.

## Introduction

Limited access to social welfare and healthcare are global challenges recognised under Sustainable Development Goal (SDG) 1 (No Poverty) and SDG 3 (Good Health and Well-Being) [53]. In Morocco, these challenges are being addressed through a shift to a consolidated social welfare model, *Registre Social Unifié* (RSU) [33]. However, the model’s implementation remains contingent on multiple structural and administrative factors which could limit full enrolment such as civic registration, literacy, geography, and institutional coordination [31]. Research shows that community-based outreach can improve enrolment in welfare programmes, especially for marginalised groups, yet community approaches remain underexplored in Morocco [14].

### Welfare models in the Global South

Contemporary welfare systems vary widely across global contexts, shaped by political histories, institutional capacity, and cultural norms. Dominant typologies of welfare models emerged from Anglo-European experiences and therefore much of the literature remains North-centric [13, 18]. In the Global South, welfare systems often arise not from consolidated bureaucratic traditions, but from legacies of colonial rule and weak state capacity [38]. These systems tend to be fragmented, divided among state, donor, and informal actors [13], targeted rather than universal [43]; heavily shaped by humanitarian frameworks [15],and framed in terms of"needs"rather than"entitlements"or “rights” [42]. Even where programmes are labelled universal, access is often conditional on documentation, geography, or eligibility assessments that can exclude marginalised groups [39]. Understanding welfare in the Global South requires recognising the hybrid forms of provision, including the socio-historic context of the social contract between the citizen and the state, interplay of geopolitical politics and donor dependency, and formal and informal social and cultural institutions shaping support systems.

### Social welfare in Morocco

Morocco’s social protection system is shaped by a centralised monarchical governance structure, influenced by the country’s strategic geopolitical position and external donor funding. This model blends Islamic charitable traditions like zakat (obligatory almsgiving) and waqf (charitable endowments) with the enduring role of dynastic authority [20, 32, 62]. As a politically stable partner in a volatile region and a key player in European Union (EU) border externalisation efforts, Morocco also attracts substantial development assistance [22]. In 2022, it received over US $1.4 billion in Official Development Assistance for social programmes, the highest in the Maghreb region: significantly more than Algeria (US $73.8 million), Libya (US $316 million), Mauritania (US $328 million), or Tunisia (US $1.29 billion) [56–60]). Within this geopolitical context, social welfare in Morocco operates not only as a response to social need but also as a mechanism for centralised state legitimacy and strategic international diplomacy [30].

Morocco faces greater social need than its neighbours with 11.6% of the population living in poverty in 2012 ($4.20 line), compared to 8.2% in Tunisia and 4.7% in Algeria [61]. However, 2020 social spending (excluding health) was 6.6% of GDP, compared to 13% in Algeria and 11.7% in Tunisia [41]. Coverage is also lower, with 42.6% of the population in Morocco covered by at least one social protection benefit (SDG 1.3.1), compared to 60.2% in Algeria, 66.1% in Libya, and 53.8% in Tunisia [29]. Together, these figures show that while Morocco faces greater social need, reported spending and coverage levels remain below those of neighbouring countries.

To improve coverage, the government is launching the Registre Social Unifié (RSU), a digital national welfare registry designed to consolidate and coordinate access to social programmes, including l’Assurance Maladie Obligatoire (AMO) (compulsory health insurance), Tayssir (conditional cash transfers for school attendance), the Direct Social Benefit programme, and the planned universal child allowance [33]. The RSU assigns each individual a unique digital identifier for civil and social protection services, records demographic and socio-economic information, and scores households based on socio-economic status to determine eligibility [33]. While the reform marks a significant step towards reducing the fragmentation of social welfare in Morocco [44], access to the RSU and its benefits remains uneven, particularly in rural areas, and is often constrained by gaps in civil registration, low literacy rates, and limited institutional coordination [31].

### Urbanisation in Souss Massa

Morocco has a population of 36.8 million, with 23.1 million living in urban areas and 13.7 million in rural regions. Rural populations face systemic inequalities, with unemployment affecting 25.9% of the population nationally and rising to 28.5% in rural areas and illiteracy affecting 19.3% of urban residents and 43.4% of rural residents [25]. These structural disparities also extend to health equity, with recent research showing unequal health outcomes across Morocco, particularly for women and marginalised groups or those requiring costly treatments like oncology drugs [7, 9]. Souss Massa, a predominantly agrarian region in Southern Morocco, has a population of approximately 3 million people, with a large rural population (39%). In recent decades, there has been rapid urbanisation in Souss Massa, with the population of the capital, Agadir, almost doubling from 254,865 in 1994 to 504,768 in 2024 [24, 25].

As in other parts of the Maghreb region, high rural-to-urban migration in Morocco is driven by climate change-induced desertification and declining agricultural opportunities [54]. Rural-to-urban migrants leave farming communities in search of work, yet many arrive in cities with limited formal education, lacking both the skills needed for urban labour markets and the extended family support structures available in their villages [8]. Additionally, navigating complex administrative processes in urban areas presents a major challenge, preventing many from accessing essential welfare services [9]. As families struggle to meet basic needs, gaps in social welfare heighten the risk of child labour and abandonment, with 1 in 650 children abandoned in 2021, contributing to an estimated 470,000 children living in institutional settings in Morocco [3, 4]. In urban centres, child homelessness and informal labour remain pressing concerns, with many children forced into hazardous work [5, 27]. Addressing these barriers is essential to ensuring that Morocco’s social welfare reforms effectively reach its most vulnerable populations, particularly in rapidly urbanising regions like Souss Massa.

### Civil registration and invisibility

Civil registration is a key administrative barrier to social programme access in Morocco. The issue disproportionately affects vulnerable groups, particularly single mothers and their children. Moroccan family law does not fully recognise legal filiation in cases of children born out of wedlock, often denying them the right to civil registration [48]. As in other parts of the Middle-East and North Africa (MENA), single mothers are reported to face discrimination when attempting to register their children, especially in cases where paternal identification is not provided or is contested [21]. Without birth certificates or family registration documents, these children are excluded from social welfare programmes, trapping families in a cycle of legal invisibility and exclusion from state services. Recent attempts at legislative reform have not fully resolved these challenges, leaving many women and children outside the protection of state social welfare [17]. These national-level reforms sit within a broader political and legal framework that shapes who is visible to the state and therefore eligible for support.

### Community-based signposting and targeting hard-to-reach populations

While existing research has examined macro-level social welfare reforms in Morocco, little attention has been given to how different delivery models can reduce exclusion among hard-to-reach groups. Research from the Global North consistently shows that hard-to-reach populations, including undocumented migrants, single mothers, and ethnic minorities, face systemic barriers to accessing care, even within well-resourced welfare states [47, 51, 64]. In the Global South, community-based, NGO-led, and peer-support strategies have been effective in bridging trust gaps and increasing service uptake [10, 35]. However, despite under-served populations facing structural barriers similar to those observed across the Global South, bottom-up community-based strategies such as NGO-led signposting remain underexplored in Morocco. This study builds on global evidence demonstrating the value of community-based models to reduce systemic exclusion and explores how these approaches can be adapted within Morocco’s semi-centralised, hybrid governance structure.

### Aims and objectives

In this study, we aimed to assess the feasibility of an NGO-led signposting model in identifying and connecting individuals with government social welfare programmes in two communes of Morocco. Our objectives were to examine the awareness of and interest in connecting with government social welfare programmes, the effectiveness of a signposting service model in facilitating enrolment, and the barriers that hindered access. We also explored the intervention implementation process and delivery challenges to inform future programme efforts to improve access to social welfare services in Morocco.

## Methods

### Design and setting

This paper forms part of the broader study, HIMAYA + (*Projet Himaya* *: Agir pour prévenir les risques & renforcer la protection des enfants en contact avec la loi*), implemented by two Non-Governmental Organisations (NGOs), Fondation Amane pour la Protection de l’Enfance (FAPE) and The Moroccan Children's Trust (MCT), and researchers from University of Auckland and Swansea University. HIMAYA + aimed to support families at risk of separation and prevent child abandonment through a multi-component community mobilisation intervention consisting of three Work Packages (WPs) [45]. WP1 facilitated participatory parenting groups for mothers involved in local community-based literacy programmes. WP2 involved conducting workshops with the general population to improve awareness of and access to government social welfare programmes. WP3 engaged local stakeholders, professionals, and government representatives through capacity-building and advocacy workshops. The overarching theory of change underpinning HIMAYA + was that increasing awareness of and guided referral to government welfare programmes would improve enrolment among vulnerable families, thereby reducing financial strain and helping to mitigate risks including child abandonment, school dropout, and informal labour. In this paper, we report on our findings from WP2 and discuss our experiences in identifying and connecting individuals with government social welfare programmes in two communes of Morocco.

HIMAYA + was delivered in the communes of Drarga and Lqliaa, located on the outskirts of Agadir, the regional capital of Souss Massa. These sites were selected due to their high levels of rural–urban migration, as identified by FAPE. Drarga and Lqliaa have populations of similar size (Drarga: 107,621; Lqliaa: 107,133) and an illiteracy rate approximately double that of Agadir (Drarga: 26.5%; Lqliaa: 30.3%; Agadir: 14.3%) [25]. The combination of high illiteracy rates, geographic location, and local knowledge indicating significant vulnerability made these communes well-suited for assessing the feasibility of an intervention aimed at improving access to government social welfare programmes for vulnerable populations.

HIMAYA + was delivered in Drarga and Lqliaa from April 2022 to December 2022, with three Social Programme Workshops (SPWs) running each week for six months from April 2022 to October 2022. We used the Template for Intervention Description and Replication (TIDieR) checklist to ensure accurate and replicable intervention description for future use [26] [Additional file 1].

### Development of guidebooks and cloud-based platform

We adopted a participatory methodology to co-design the study. This process began with co-creating two guidebooks and a digital case management system to assist staff in implementing the study and facilitate the organisation and analysis of study and process outcome data [Additional files 2 and 3]. The first guidebook incorporated feedback from service users, frontline staff, management, and executive team from FAPE and senior staff from MCT, as well as drawing upon existing resources [11, 40, 55]. FB initially co-created guidebooks in English, which AS then translated to Arabic. Guidebooks were then iteratively refined with FAPE staff over multiple workshops, where they provided feedback on structure, language and translation quality, and the relevance and accuracy of the content. FB in collaboration with staff from FAPE co-developed a digital case management system to manage all project and research data which was hosted on Google Drive. The digital management system could be used in Arabic, English, or French, and staff were encouraged to enter information in their preferred language.

### Participants and recruitment

We recruited and trained eight local staff members for the HIMAYA + project. Staff contacted the directors of all 44 schools across the communes of Drarga and Lqliaa inviting them to participate in the study. Staff then presented our study to the parents’ association attached to each participating school and asked them to share a flyer with all parents at the school inviting them to attend a Social Programme Workshop (SPW). We also provided local NGO staff with information about the SPWs which they passed on to their service users via word-of-mouth and WhatsApp. Permission was also obtained from local authorities for our study staff to erect a tent in prominent areas of Drarga and Lqliaa, where staff members could be approached by the public to learn about the available government social welfare programmes. In this paper, we will report data from the SPWs conducted at schools. The SPWs at schools were chosen to ensure our sample was homogenous, avoiding the variability of participants from open cohorts at tents or pre-identified NGO referrals.

### The four-stage process for intake and referral

**Fig. 1:**
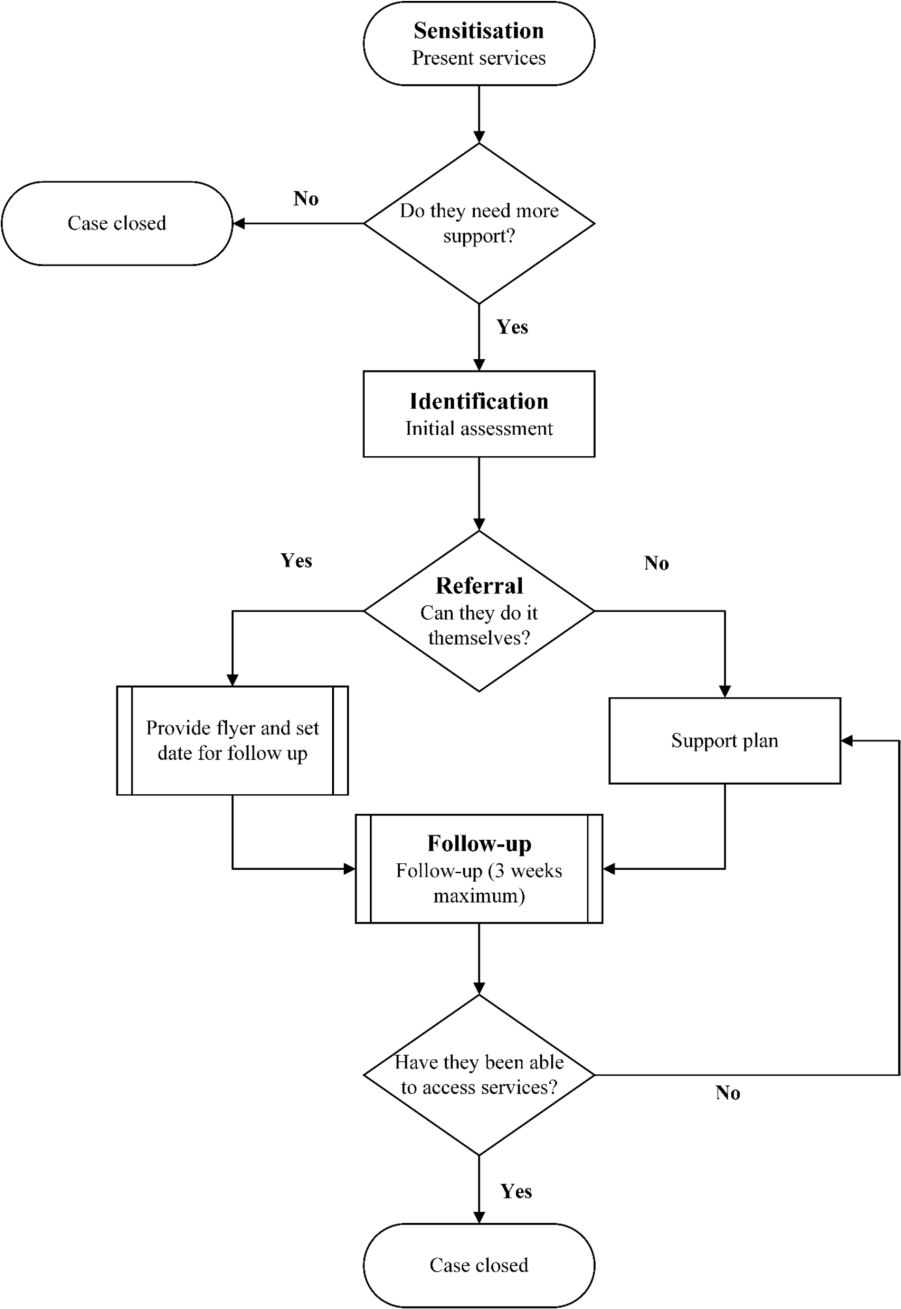
This figure illustrates the stages of our community mobilisation approach as a flow chart

Our intervention design was grounded in a theory of change guided by the community mobilisation model [45]. This model posits that increasing awareness of social welfare services, combined with guided referral and follow-up support, would improve enrolment and health and social outcomes among vulnerable families. Improved access to services – particularly cash transfers, healthcare coverage, and civil registration – was expected to alleviate household financial strain and reduce the risk of adverse child outcomes, including child abandonment, school dropout, and exposure to informal labour. This model also informed the four-stage delivery approach used in the SPWs (Fig. 1).

In the sensitisation stage, frontline staff conducted SPWs in schools and community spaces to inform participants about key government social welfare programmes. These included the Régime d’Assistance Médicale (RAMed) for subsidised healthcare, Tayssir, a conditional cash transfer programme for school attendance, and civil registration, a prerequisite for accessing government benefits. Frontline staff delivered information through oral presentations, printed materials, and interactive discussions. Facilitators encouraged participants to ask questions, raise concerns, and share their previous experiences attempting to access support.

During the identification stage, frontline staff conducted structured needs assessments using a standardised intake form. This form recorded household composition, current access to social welfare, and specific barriers to enrolment. Families without civil registration were identified as high-priority cases due to the systemic barriers they faced in accessing services and the anticipated length of time required to support them.

The referral stage involved connecting families with the appropriate service providers and guiding them through application procedures. Frontline staff provided step-by-step instructions, facilitated document collection where necessary, and, in cases requiring additional support, liaised directly with local agencies. Families then received individualised follow-up plans to track their enrolment progress.

The follow-up stage ensured that families successfully completed the enrolment process. Frontline staff conducted follow-up calls and in-person visits within three weeks of initial referral to assess whether participants had accessed services. If any additional challenges were identified, frontline staff conducted additional consultations with families and local agencies.

More information on the modalities we adopted and the implementation and recording of different aspects of the intervention day-to-day can be found in the guidebooks we provided to frontline staff (Additional files 1, 2).

### Focus groups and interviews

We conducted a focus group with seven study frontline staff, and an interview with the study manager and study director at the completion of the study period to gain feedback on their experiences delivering the study. FB developed focus group and interview guides in English and AS translated them into Arabic. A FAPE staff member independent from the HIMAYA + study team conducted the focus groups with frontline staff and the interview with the study manager in Darija. FB conducted the interview with the study director in English. The audio from focus groups and interviews was recorded. FAPE staff independent to the HIMAYA + study team then transcribed audio recordings verbatim into Arabic. AS translated the Arabic transcripts into English, with clarity in translation provided by FB.

### Data analysis

#### Study and process outcomes

We defined process outcomes with our study funders at the project’s outset to include the number of SPWs held, participant demographics, numbers of attendees, types of support requested and achieved, as well as eligibility and reasons for ineligibility for government social welfare programmes. Quantitative data were recorded and analysed in Google Sheets using descriptive statistics [2]. We followed Medical Research Council guidance on developing complex interventions and report on the feasibility, reach, and acceptability of our intervention [50]. We assessed feasibility by comparing planned versus delivered SPWs and monitoring delivery of our four-stage process. We evaluated reach by examining the proportion and demographic characteristics of participants relative to our target population (20% attendance rate). We indirectly measured acceptability via participant retention and successful follow-up (> 60% successful referrals), providing insight into families’ willingness to engage with the intervention.

#### Qualitative data

We analyse the data deductively using thematic analysis [6]. FB coded all transcripts using our four stages of intake and referral – “Sensitisation”, “Identification”, “Referral” and “Follow-up” – as the primary codes. Data that did not fit these categories were coded under an additional theme “staff training and management of study data”. AS and CH provided feedback on final themes to ensure consistency and clarity in coding. Quotations from focus groups are identified as Frontline Staff by letters then a number identifying the respondent (e.g. FS-1), quotations from the manager or director are named as such.

## Results

### Social programme workshops: participation and outcomes

The broader intervention engaged a total of 3,958 individuals across three delivery settings: (1) school-based Social Programme Workshops (SPWs) (*n* = 1,087), (2) mobile tents based in public spaces (*n* = 2,416), and (3) sessions at local NGO offices (*n* = 455). However, due to methodological inconsistencies in recruitment and data collection at tent and NGO venues, this analysis reports only on the school-based SPWs.

We conducted 18 school-based SPWs, reaching 1,087 of the 5,425 (20%) members of 16 parents’ associations across 18 of the 44 schools in Drarga and Lqliaa. Participating schools included 11 primary schools, four collèges, and one lycée (Table 1). The highest proportion of parents attending a single workshop was 44%, and the lowest was 4%. This variation reflected differences in school engagement, visibility of outreach, and timing of authorisation.

**Table 1:** This table details the number of students, Parents’ Association (PA) members, female and male attendees, total attendees, and percentage of PA members who attended across various schools and colleges

School	Students	PA* members	Female attended	Male attended	Total attended	% of PA attended
École Primaire Aknibich	825	180	28	19	**47**	26%
École Primaire Al Firdaous	1384	365	94	4	**98**	27%
École Primaire Al Hidaya	1244	335	7	14	**21**	6%
École Primaire Al Izdihar	1261	341	5	58	**63**	18%
École Primaire Al Mouahidine	745	305	9	4	**13**	4%
École Primaire Al Yamama	788	343	34	5	**39**	11%
École Primaire Amina Bintou Ouaheb	820	352	70	6	**76**	22%
École Primaire Aouina	1526	365	59	5	**64**	18%
École Primaire Eguidar Al Jadida	784	310	42	14	**56**	18%
École Primaire Elhansali	1168	262	36	7	**43**	16%
École Primaire Oued Souss	1776	523	87	33	**120**	23%
Collège Al Bayrouni	1088	233	49	0	**49**	21%
Collège El Hansali	989	273	28	1	**29**	11%
Collège El Yassamine A	1473	411	6	0	**6**	1%
Collège El Yassamine B	1473	411	15	2	**17**	4%
Collège Lagouira A	1813	582	37	4	**41**	7%
Collège Lagouira B	1813	582	93	105	**198**	34%
Lycée Errazi	229	245	40	67	**107**	44%
**Total**	17,913**	5,425***	**739**	**348**	**1,087**	**20%**

Attendance at social programmes workshops

^*^ PA = Parents’ Association

^**^ We have subtracted the number of students from the same schools

^***^ We have subtracted the number of parents from the same schools

Higher attendance was generally observed in schools with stronger parent–school relationships and earlier mobilisation. For example, after low turnout at two initial SPWs, we re-engaged the relevant parents’ associations and held follow-up sessions with improved participation. On average, each SPW had 60 participants, with 74% female and 26% male attendees; two workshops included only female participants.

**Fig. 2:**
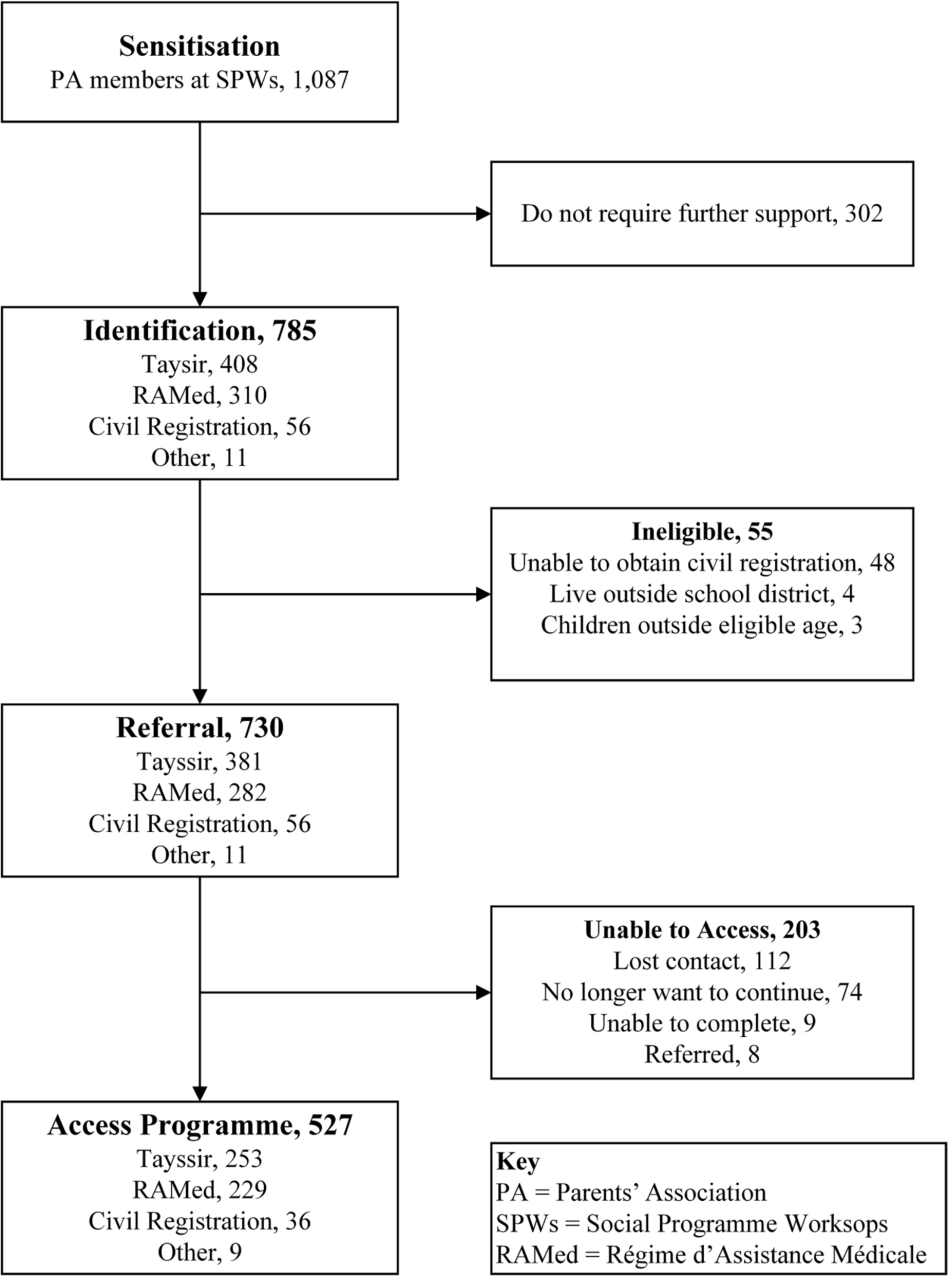
This flowchart details the number of individuals who were referred, successfully registered, and subsequently benefited from the programmes

During the SPWs, 785 (70%) attendees requested support to access government social programmes (Table 2). In Drarga, most sought assistance with Tayssir (60%), whereas in Lqliaa, slightly more requested RAMed (54%). Civil registration was the least requested service across both sites (5% in Drarga; 10% in Lqliaa). However, a lack of valid civil documents – particularly expired or missing national identity cards – emerged as a key barrier during follow-up, preventing some parents from enrolling in RAMed or Tayssir (Fig. 2). Parents also requested support for other services, including disability assessments, specialised healthcare, and the government widows’ support scheme, which were grouped under “other”.

**Table 2:** This table lists the social programmes that were requested by participants during the study, including civil registration, RAMed (subsidised healthcare), and Tayssir (conditional cash transfer for education)

Location	Total	RAMed	Tayssir	Civil Registration	Other
Dragra	529	173 (32.7%)	322 (60.9%)	28 (5.3%)	6 (1.1%)
Lqlilla	256	137 (53.5%)	86 (33.6%)	28 (10.9%)	5 (2.0%)
**Total**	785	310 (39.5%)	408 (52.0%)	56 (7.1%)	11 (1.4%)

Social Programmes Requested

Of the 785 parents who requested support, 55 (5%) were deemed ineligible for government social programmes. Most of these cases (87%) were linked to civil registration challenges that could not be resolved within the scope of the project. While the project offered support to obtain or renew civil registration, some families faced barriers such as the lack of documentation, legal disputes, or the need to travel long distances to their place of birth. These cases were classified as ineligible, as staff were unable to assist participants in fulfilling the required administrative or legal procedures. A smaller proportion were ineligible for Tayssir due to residing outside the school district (7%) or having children outside the eligible age range (5%).

Of the eligible participants, 67% were successfully connected to their desired government programme. The highest rates of successful referral were observed for RAMed (81%) and other services (82%), followed by Tayssir (66%) and civil registration (64%).

The project was unsuccessful in connecting 28% of eligible parents to their desired programme. The highest proportions of unsuccessful cases were related to Tayssir (34%) and civil registration (36%). The most common reason for unsuccessful referrals was a loss of contact; 55% of these participants had disconnected phone numbers or did not respond to follow-up calls. Other reasons included families choosing not to continue (36%), referrals being redirected to more appropriate providers (4%), or an inability to complete the necessary procedures despite eligibility (4%).

### Facilitators and barriers to civil registration

Frontline staff reported that civil registration was often the first issue that needed to be addressed before families could access programmes like RAMed or Tayssir. However, the process was time-intensive and often exceeded the project’s follow-up period. As one staff member explained, “registering birth records takes a lot of time and effort, leaving little room to address RAMed needs” (FS-4). Others highlighted challenges in maintaining contact with participants, noting that “sometimes their phones don’t work, and we might need to extend the tracking time to reach all the vulnerable cases” (FS-2).

Travel was also a common obstacle, particularly for participants born in distant provinces. In some cases, accessing the necessary documents required extended delays. “First, they say their birth certificate is far away and they need to travel to get it,” explained one staff member, “then, you wait more weeks to gather the rest of the documents. After that’s sorted, you start persuading them to apply for the RAMed card” (FS-3). In more complex cases, participants abandoned the process altogether. One example involved a single mother who, according to staff, “couldn’t travel to Casablanca to get the documents and register her children. She had no money and couldn’t take her children with her. What could we do? We could not continue” (FS-5).

Some cases were further complicated by inconsistent requirements across civil status offices, especially when paternity needed to be established. One staff member described how procedures varied between officers:

If the documents are old or you have an old ID [civil registration] card, they can register you without any problem. But another officer might say you need to bring the child, check if you are the father or not, and if you fail, you lose 500 dirhams just to find this out. (FS-1)

Even when mediation was attempted, outcomes were uncertain. In one case, staff sought support from inspectors in Agadir at the provincial level, but the request was still refused, despite an initial payment to begin the process. “It still cost us 700 dirhams, and nothing moved forward,” explained FS-4.

### Gaps in agency coordination

Staff encountered difficulties engaging with government services, largely due to the lack of legal recognition for social workers. Without formal authority, they were sometimes dismissed by officials. “Even if you go with the person,” one noted, “they [government officials] might not cooperate with you and ask who you are, what your authority is” (FS-3). The study director echoed this, pointing to the absence of a national framework: “We lack a clear legal framework or guidelines for social work – a statute. That leaves everything open to interpretation”.

Inconsistent requirements across agencies created further delays. Staff explained that in some cases, if participants didn’t have the right documents, officials would give them a hard time without offering practical solutions and “just keep explaining the procedures to them” (FS-2). In response, staff often needed to accompany families in person to ensure they understood the steps required. This support made a difference, with one staff member observing, “when the person goes alone, officials always reject their request, but when accompanied by a social worker, more officials respond, and they face fewer difficulties” (FS-4).

As the programme progressed, several staff members observed improved collaboration from local actors. “Even the officials at the municipalities and schools contacted us and informed us that there is a case, and we should help them register,” said a frontline staff member, explaining, “They came from all over the place” (FS-1). The study director added that these relationships helped “bridge gaps and enhance coordination among existing actors,” contributing to more effective support for families.

### Process challenges to implementing the workshops

Frontline staff encountered multiple challenges during the rollout of the Social Programme Workshops (SPWs), particularly in securing authorisation for school-based activities. These delays affected delivery, with workshops ultimately taking place in only 16 of the 44 schools initially contacted. In many cases, school directors expressed the need for formal clearance from the regional Education Department before proceeding. As one staff member explained,

From the beginning, any director we spoke with said that an authorisation from the Education Department was necessary, and we didn’t have the right to talk with the department at that level, so we couldn’t get the authorisation. (FS-1)

In a few instances, previously granted permissions were later revised, sometimes shortly before scheduled workshops.

Despite these disruptions, project managers reflected positively on the decision to focus efforts on primary schools. This decision aligned with emerging needs on the ground. “With schools, especially primary schools, there are a lot of children that might not be officially registered,” noted the study director, “So it was good we chose them”. The volume of participation exceeded expectations, with the study reaching far more families than initially planned. “Our target was 300 cases, having to support 800 families,” said the study manager. “We far exceeded our expectations, reaching 2,200–2,300 families, and having so many at the primary schools was a big reason”.

### Training and management

Staff described the opportunity to shadow professionals from FAPE as a particularly valuable element of their preparation. One frontline worker shared that they had.

Spent a day at the centre and shadowed a social worker responsible for civil registration. I accompanied him to various places like hospitals and prisons, learning communication skills and how to work with different actors. (FS-7)

Having a point of contact for questions during the project was also appreciated by the same staff member, “it was very helpful to be able to frequently seek his advice”.

However, some staff felt the initial training was too theoretical and lacked the practical depth needed for the realities of implementation. According to the study manager, “at the project’s start, we realised the training wasn’t enough. We only truly learned during the project, uncovering many unclear areas”. Another team member echoed this sentiment, noting, “we worked a lot on the theoretical part in training but missed the practical” (FS-6). In contrast, the guidebooks developed for the project were widely seen as useful. “The guide was essential for our work in the field,” said one participant:

It provided all the necessary information and helped us correct any mistakes we made. It contained instructions for every step of our work, from social investigations to managing support for individuals. (FS-2)

Data management was another area that received mixed feedback. While the shared cloud-based system helped coordinate tasks and track progress, data entry was time-consuming. As the study manager explained,

Sometimes we recorded the same information in multiple documents, which wastes time and effort for social workers. It would be more efficient if we only recorded it once, especially for civil registration cases.

## Discussion

This study assessed the feasibility of an NGO-led signposting model in linking individuals with government social programmes in two Moroccan communes. The intervention enhanced awareness of available support, although enrolment was sometimes hindered by eligibility criteria and administrative hurdles. The NGO-led signposting model demonstrated promising potential by bridging service access gaps, despite challenges related to bureaucracy, unclear documentation, and limited follow-up. While the study’s short-term, site-specific nature limits generalisability of broader impacts, our findings underscore the value of an NGO-driven identification and referral service. Future efforts could build on this intervention to streamline access, strengthen coordination, partner with local community-based NGOs, and assess the intervention at scale across Morocco as it aims to transition to the consolidated *Registre Social Unifié* (RSU) by 2030.

### Evidence on community-driven welfare enrolment strategies: how our findings compare

#### Awareness and demand for government welfare programmes

Limited awareness of social welfare programmes among those who need them most is a persistent challenge in global health systems [37]. In our study, 72% of participants expressed a desire to be connected to government welfare programmes, yet prior to engagement, they were unaware of their eligibility or how to navigate the application processes. This mirrors global findings that link low awareness to structural barriers such as lack of knowledge of services and bureaucratic complexity [19]. Studies in other Low- and Middle-Income Countries (LMICs) have demonstrated that marginalised populations, particularly those with lower literacy levels or rural residency, are least likely to seek out and benefit from social protection schemes (Bright, 2017). In Morocco, limited awareness of welfare support has been reported in relation to RAMED – now AMO – with many eligible families failing to enrol due to misconceptions or lack of outreach [1]. These findings underscore the need for targeted awareness-raising efforts, ensuring that information reaches those who need it most through accessible and community-driven mechanisms.

#### Signposting service model in facilitating enrolment

NGO-led signposting services can play a significant role in bridging the gap between government welfare programmes and eligible beneficiaries, especially for hard-to-reach and marginalised groups [16]. In our study we successfully enrolled 67% of the parents who requested support in a government social welfare programme, demonstrating the effectiveness of our model. This aligns with findings from other settings where NGOs serve as essential allies to governments by raising awareness, providing application assistance, and facilitating referrals [34]. Research in LMICs highlights that NGOs are particularly effective in engaging hard-to-reach populations, often employing community-based mobilisation strategies to ensure participation, comprehension, and delivery of social programmes [14]. In Morocco, NGOs have played a key role in facilitating access to social welfare programmes, yet their involvement remains ad hoc rather than institutionalised [65]. Formalising partnerships between NGOs and government agencies could enhance enrolment efforts, ensuring sustainable and structured pathways to access.

#### Barriers that limited access

While civil registration was one of the focal services offered through the intervention, it also functioned as a precondition for accessing other programmes. This dual role created a structural barrier; some families who wished to enrol in RAMed or Tayssir were deemed ineligible due to missing civil documentation (e.g., birth certificates). For some families, our team was able to facilitate access to civil registration, but for others, particularly those lacking proof of parental identity or facing legal disputes, the process remained unresolved at the end of the study period. This barrier is well-documented in the wider Middle-East and North Africa region, where legal identity requirements are reported to prevent vulnerable populations from accessing healthcare, education, and social assistance [21]. Additionally, the frontline workers in our study reported that the absence of a legal statute for social workers limited their capacity to intervene effectively in these cases. Similar challenges have been reported in the management of chronic diseases such as type 2 diabetes in Morocco, where health workers cited excessive workload, supply interruptions, weak referral pathways, and insufficient training as key barriers to equitable service delivery [12]. Simplifying civil registration processes and formally recognising the role of social workers in supporting vulnerable populations could help overcome administrative hurdles.

#### Intervention implementation

A key strength of the HIMAYA + intervention was its participatory design, which included ongoing communication with management, staff, and former service users of FAPE to facilitate continuous real time co-design. While guidebooks and tracking systems enhanced efficiency, staff struggled with some duplication in recording instruments and the need to balance recording their work with the *doing* of the work. While participatory approaches can ensure contextual relevance, they do require continuous adaptation to be effective [49]. The Medical Research Council (MRC) framework for complex interventions highlights the need for iterative refinement, ensuring interventions remain flexible and responsive [50]. Future participatory interventions should ensure interventions evolve in response to frontline workers’ experiences.

### Expanding NGO-led signposting models in Morocco

Our pilot indicates that an NGO-led signposting model can be an effective mechanism for supporting access to social welfare, particularly in vulnerable communities. The intervention was feasible, successfully delivering all planned Social Protection Workshops (SPWs) over six months and implementing the four-stage process of sensitisation, identification, referral, and follow-up. Despite only conducting SPWs at 36% of the schools contacted across Drarga and Lqliaa, the programme achieved broad reach and strong engagement, particularly within communities with illiteracy rates nearly twice those of the regional urban centre. We attribute the limited school uptake not to flaws in the intervention itself, but rather to structural issues, such as the limited authority of social workers and the absence of a formal social work statute. The high demand for support (70% enrolment requests) and a 67% successful linkage rate, suggests potential community acceptability and the model’s practical value. These outcomes suggest higher than average reach and engagement, particularly when compared to national coverage levels, where government medical coverage was estimated to be a combined 60% (30% AMO, 30% RAMed) in 2019 [63]. Our findings suggest that NGO-led signposting may complement national efforts by improving uptake among vulnerable and hard-to-reach communities.

While the intervention demonstrated feasibility in two communes in Southern Morocco, the model’s transferability to other settings requires careful consideration. Theunissen et al. [52] highlight how participatory models need adapted tools, language, and intermediaries to avoid deepening inequities in access and service delivery. Key enabling factors in this study included: the presence of an established NGO with long-standing community trust; access to school infrastructure for outreach; strong relationships with local authorities; and the ability to draw on expert tailored knowledge to create guidebooks and databases and train local frontline staff. In settings without these foundations adaptations may be necessary, such as in remote rural areas [23] or conflict-affected zones [28]. For example, digital platforms or mobile outreach units may be required where access is limited and partnerships with faith-based or informal community leaders may substitute for NGOs [36]. Further, legal ambiguity around social work roles may present greater challenges in regions where public officials are less responsive to community actors [46]. Future implementation studies should therefore assess the contextual preconditions for replicability, including local knowledge and skills, sustained access to tailored support, governance environments, social infrastructure, and institutional trust.

### Strengths and limitations

Our study’s key strength lies in its community-based and participatory approach. By engaging directly with parents, frontline social workers, and community stakeholders, we ensured the intervention was contextually relevant. The SPWs provided a structured yet flexible platform for information-sharing and enrolment support, demonstrating the potential of NGO-led initiatives to improve access to welfare services. While the study reached nearly 4,000 individuals, only data from school-based implementation were included due to inconsistent recruitment procedures. This may under-represent the experiences of excluded groups such as out-of-school children or those with mobility constraints.

We were unable to access official government data on enrolment rates in the target social programmes within the study communes, which limited our ability to contextualise the intervention's reach relative to local baseline enrolment levels. Combined with the absence of a comparison group, this limited our ability to attribute enrolment success solely to the intervention nor assess whether a government-led or alternative delivery approach would have been more effective. While we included service user of FAPE in the design of the guidebooks, we did not conduct interviews or focus groups with beneficiary families of this study, which due to ethical and resource constraints were not feasible during this phase but should be prioritised in future work to capture service users' experiences more directly. Additionally, the study’s scope was restricted to two communes, which may not fully reflect the challenges faced in other regions with different socioeconomic or administrative conditions. Lastly, the study's duration of six months also posed limitations, as not all referred cases could be followed up, highlighting the need for long-term studies to track registration outcomes. However, the intervention was still able to reach a relatively a large population and facilitate access to key services within this short timeframe and limited resources, suggesting strong potential for scale.

## Conclusions

The Moroccan government has introduced evidence-based programmes aimed at reducing health inequity by making welfare more accessible through a consolidated digital system. However, our findings indicate that uptake remains limited in the very communities these reforms are intended to reach. The rollout of the Registre Social Unifié (RSU) may help address this gap, but persistent legal and administrative barriers, particularly around civil documentation, continue to impede access. Our study suggests that an NGO-led signposting strategy can complement national efforts by facilitating awareness, guided referrals, and follow-up. Yet without structural reforms such as simplifying civil registration, formally recognising the social work profession, and improving inter-agency coordination, these strategies risk compensating for, rather than transforming, systemic fragmentation. This, in turn, may deepen the invisibility of the most disadvantaged groups such as single mothers and their children and those living in rural and remote areas. Future research should examine the RSU’s impact on access, assess how legal frameworks shape frontline delivery, and explore pathways for embedding NGO-led models within Morocco’s evolving welfare landscape.

## Supplementary Information

Additional file 1. This file contains the TIDieR (Template for Intervention Description and Replication) checklist, detailing the essential elements of our intervention to ensure transparency and replicability of the study

Additional file 2. This guidebook outlines the Five Pillars of our project: Holistic Approach, Forming Relationships, Transition Support, Child-Centred Decision Making, and Professionalism

Additional file 3. This guidebook details our referral process and the tools used for recording, assessing, and following up on referrals, including the Work Journal, Initial Assessment, Referral Tracking Document, and Case Journal

## Data Availability

No datasets were generated or analysed during the current study.

## Abbreviations

AMO: L’Assurance Maladie Obligatoire
FAPE: Fondation Amane pour la Protection de l’Enfance
GDP: Gross Domestic Product
HIMAYA +: Projet Himaya + : Act to prevent risks & strengthen the protection of children in contact with the law
LMICs: Low- and Middle-Income Countries
MCT: The Moroccan Children's Trust
MENA: Middle-East and North Africa
NGO: Non-Governmental Organisation
PA: Parents Association
RAMed: Régime d’Assistance Médicale
RSU: Registre Social Unifié
SPWs: Social Programme Workshops
WPs: Work Packages
