# Supplementary material for: Piloting a NGO-led signposting intervention to improve access to government welfare in Southern Morocco: a feasibility study
Source: Int J Equity Health. 2025 Oct 16;24:280. doi: 10.1186/s12939-025-02605-0 (PMC12532411; doi:10.1186/s12939-025-02605-0)
Supplement: Supplementary file 1 — Additional file 1. This file contains the TIDieR (Template for Intervention Description and Replication) checklist, detailing the essential elements of our intervention to ensure transparency and replicability of the study [file 12939_2025_2605_MOESM1_ESM.docx]

**
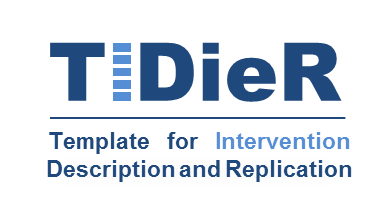
The TIDieR (Template for Intervention Description and Replication) Checklist*:**

Information to include when describing an intervention and the location of the information

| **Item number** | **Item** | **Where located **** | |
| --- | --- | --- | --- |
|  |  | Primary paper  (page or appendix  number) | Other ^†^ (details) |
|  | **BRIEF NAME** |  |  |
| **1.** | Provide the name or a phrase that describes the intervention. | _____2.1.____ | The intervention is referred to as “Social Programme Workshops (SPWs)” and is highlighted in the title and abstract. It’s also described as one part of a large multi-component community mobilisation intervention. |
|  | **WHY** |  |  |
| **2.** | Describe any rationale, theory, or goal of the elements essential to the intervention. | ____1.3.,1.4______ | The rationale is provided in the Introduction and Background sections, linking improved awareness and enrolment in social programmes to better access and reduced vulnerability. |
|  | **WHAT** |  |  |
| **3.** | Materials: Describe any physical or informational materials used in the intervention, including those provided to participants or used in intervention delivery or in training of intervention providers. Provide information on where the materials can be accessed (e.g. online appendix, URL). | _____2.2._____ | The manuscript mentions the use of two guidebooks (developed in English and translated into Arabic) and a digital cloud-based case management system. |
| **4.** | Procedures: Describe each of the procedures, activities, and/or processes used in the intervention, including any enabling or support activities. | ___2.3., 2.4. ____ | A four-stage process is described (sensitisation, identification, referral, follow-up) that outlines the operational steps during the SPWs. |
|  | **WHO PROVIDED** |  |  |
| **5.** | For each category of intervention provider (e.g. psychologist, nursing assistant), describe their expertise, background and any specific training given. | _____2.3._____ | Local staff were recruited, trained, and deployed to conduct the SPWs. Brief roles are mentioned. |
|  | **HOW** |  |  |
| **6.** | Describe the modes of delivery (e.g. face-to-face or by some other mechanism, such as internet or telephone) of the intervention and whether it was provided individually or in a group. | ___2.3., 2.4.___ | The intervention was delivered in-person through workshops held in school settings and community areas. |
|  | **WHERE** |  |  |
| **7.** | Describe the type(s) of location(s) where the intervention occurred, including any necessary infrastructure or relevant features. | ______2.3______ | SPWs were conducted in schools within two Moroccan communes (Drarga and Lqliaa) on the outskirts of Agadir. |
|  | **WHEN and HOW MUCH** |  |  |
| **8.** | Describe the number of times the intervention was delivered and over what period of time including the number of sessions, their schedule, and their duration, intensity or dose. | ___2.3, 3.1____ | workshops were held weekly for six months (April 2022 to October 2022) with three sessions per week. |
|  | **TAILORING** |  |  |
| **9.** | If the intervention was planned to be personalised, titrated or adapted, then describe what, why, when, and how. | ___2.1. _____ | The intervention was not tailored for specific communities but due to the participatory methodology used was co-design with relevant stakeholders from the delivering local institution. |
|  | **MODIFICATIONS** |  |  |
| **10.^ǂ^** | If the intervention was modified during the course of the study, describe the changes (what, why, when, and how). | _____3.1._____ | The only modification was the due to low turnout at two initial SPWs, we recontacted the parents’ association of the two schools requesting more attendees |
|  | **HOW WELL** |  |  |
| **11.** | Planned: If intervention adherence or fidelity was assessed, describe how and by whom, and if any strategies were used to maintain or improve fidelity, describe them. | ___2.6.__ | The digital case management system was introduced as a tool to monitor adherence to the four-stage process, and process outcomes were planned to be recorded. |
| **12.^ǂ^** | Actual: If intervention adherence or fidelity was assessed, describe the extent to which the intervention was delivered as planned. | ____3.1._____ | Process outcomes such as the number of participants, percentage receiving enrolment support, and success rates for connecting to programmes are reported. |

** **Authors** - use N/A if an item is not applicable for the intervention being described. **Reviewers** – use ‘?’ if information about the element is not reported/not sufficiently reported.

† If the information is not provided in the primary paper, give details of where this information is available. This may include locations such as a published protocol or other published papers (provide citation details) or a website (provide the URL).

ǂ If completing the TIDieR checklist for a protocol, these items are not relevant to the protocol and cannot be described until the study is complete.

* We strongly recommend using this checklist in conjunction with the TIDieR guide (see *BMJ* 2014;348:g1687) which contains an explanation and elaboration for each item.

* The focus of TIDieR is on reporting details of the intervention elements (and where relevant, comparison elements) of a study. Other elements and methodological features of studies are covered by other reporting statements and checklists and have not been duplicated as part of the TIDieR checklist. When a **randomised trial** is being reported, the TIDieR checklist should be used in conjunction with the CONSORT statement (see [www.consort-statement.org](http://www.consort-statement.org)) as an extension of **Item 5 of the CONSORT 2010 Statement.** When a **clinical trial** **protocol** is being reported, the TIDieR checklist should be used in conjunction with the SPIRIT statement as an extension of **Item 11 of the SPIRIT 2013 Statement** (see [www.spirit-statement.org](http://www.spirit-statement.org)). For alternate study designs, TIDieR can be used in conjunction with the appropriate checklist for that study design (see [www.equator-network.org](http://www.equator-network.org)).
