# Supplementary material for: Piloting a NGO-led signposting intervention to improve access to government welfare in Southern Morocco: a feasibility study
Source: Int J Equity Health. 2025 Oct 16;24:280. doi: 10.1186/s12939-025-02605-0 (PMC12532411; doi:10.1186/s12939-025-02605-0)
Supplement: Supplementary file 2 — Additional file 2. This guidebook outlines the Five Pillars of our project: Holistic Approach, Forming Relationships, Transition Support, Child-Centred Decision Making, and Professionalism [file 12939_2025_2605_MOESM2_ESM.docx]

#
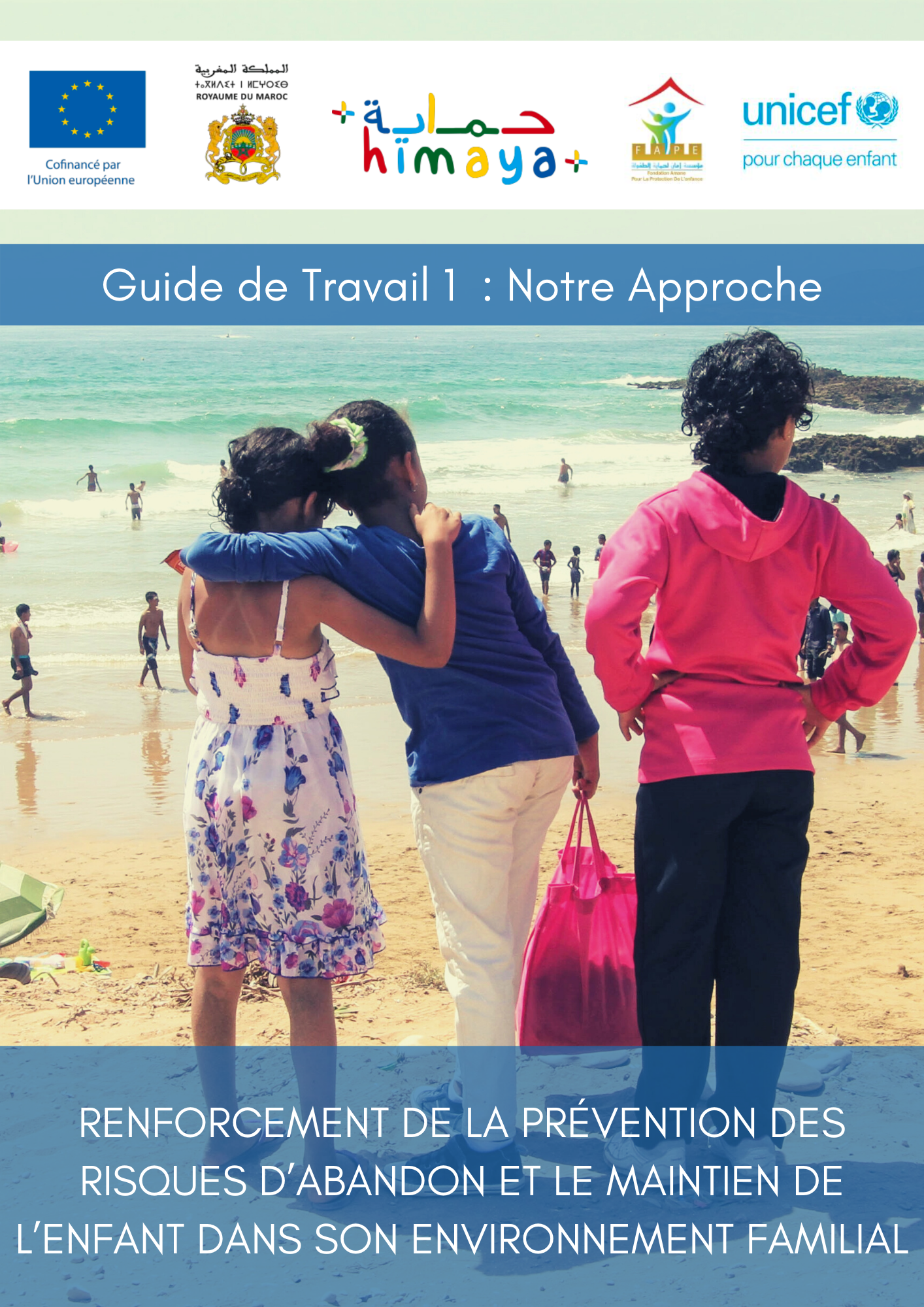


# Table of Contents

[Using The Four Stages Guidebooks 1](#_Toc94517467)

[1. Holistic 2](#_Toc94517468)

[Acknowledging Intersections 2](#_Toc94517469)

[The Social Service Team 3](#_Toc94517470)

[Collaboration 5](#_Toc94517471)

[2. Relational Social Work 6](#_Toc94517472)

[Engagement 6](#_Toc94517473)

[Communication: An Empathetic Approach 8](#_Toc94517474)

[Trauma, Attachment and Transference 8](#_Toc94517475)

[Trauma 9](#_Toc94517476)

[Attachment Theory 9](#_Toc94517477)

[Transference 10](#_Toc94517478)

[3. Transitional 12](#_Toc94517479)

[Entry and Exit Points 12](#_Toc94517480)

[Participatory 2](#_Toc94517481)

[Strengths-Based Solutions Focused 2](#_Toc94517482)

[What does strengths-based mean? 2](#_Toc94517483)

[Circles of Support 3](#_Toc94517484)

[4. Child-Centred 5](#_Toc94517485)

[Being Child-Centred and Family-Focused 5](#_Toc94517486)

[Child-Centred 5](#_Toc94517487)

[Family Focused 5](#_Toc94517488)

[5. Professional 7](#_Toc94517489)

[Vision and Purpose 7](#_Toc94517490)

[Confidentiality 7](#_Toc94517491)

[Accountability and Transparency 8](#_Toc94517492)

## Using The Guidebooks

The following guides are that, a guide, and a guide only. They are designed to provide frontline staff and management a guide to effectively delivering and understanding the ways we understand and implement the project**.** This first guidebook provides an understanding of the approach used in our project to deliver effective and sustainable services. This approach is underpinned by 5 Pillars which are embedded into our assessment process and practiced in our everyday work with children and families, other child protection actors, and internally amongst and between staff and management. Understanding the ways the 5 Pillars encompass and are embedded into the logic of practice of the work enables all workers and managers to be able to speak the same language and have a common purpose when developing plans in unforeseen circumstances. The 5 Pillars will be used in the following guidebooks as a way of defining our practice through practice. The 5 Pillars are:


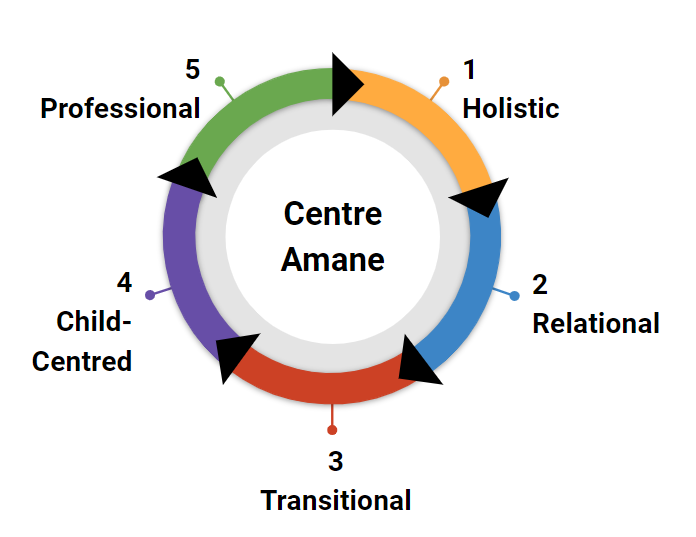


Each pillar is not isolated, rather all 5 Pillars are required to continuously be used together – e.g. to be **holistic**, we need to form **relationships**, ensure children and families are able to **transition** through our services, keep the rights of the **children at the centre** of decisions, and maintain **professionalism** by practicing confidentially, accountability, and transparency.

It’s important to remember our **limits** as care providers in this timeframe. A good tip is to keep in mind that our project is about **building the capacity** of both the children and families we are supporting and the local child protection professionals. Therefore, you must always ensure to not commit to providing support to children and families we are unable to fulfill or provide support that local professionals will be better equipped to provide. The 5 Pillars are here to guide you to balance providing support and not overcommitting yourself and the wider team. However, it also recommended to always consult with your fellow team members and manager in formal supervisions and in daily team meetings and informal communications.

# Holistic

Holistic social work is about being aware of the ways the social environment surrounding children and families affects the mode of care delivery. To maintain our holistic approach, we ensure to:

- recognise the inter-related aspects of each individuals life in order to continuously reflect on the ways the lives of children and families change and adapt to new circumstances
- acknowledge that no single organization or person is able to respond to every aspect of an individual's life
- gather and evaluate information from different sources

Being holistic is not about compartmentalising the many parts of an individual or only for the different services which are to be delivered. Being holistic in practice and thinking is something that should be performed continuously by each team member and the team every day. It is infused in our practice, logic, and ways of thinking about our work and the delivery of services. Likewise, **the 5 Pillars are to be understood “holistically” rather than in isolation**.

## Acknowledging Intersections

Taking a holistic approach requires service providers to look beyond the child and families presented self. It requires an acknowledgement and recognition of the *intersections* of each individual's life and the ways they interact in a variety of social, cultural, economic, and political structures. Intersectionality is a way to understand the different “intersections” between, within, and outside someone's life which contribute to the person and circumstances presented.

A good exercise is to look at the figure on the next page and place **your** intersections into the above diagram before asking yourself:

- Does knowing my gender tell someone everything they need to know about me?
- Does knowing my current profession explain why I entered the profession, why I am still in the profession, and the realities of my everyday life?
- Does my level of education define how smart I am?
- Does how much money I have illustrate my success in life or are there other factors at play?
- In what ways does the neighbourhood I live in reflect who I am? What about the bed I sleep in? The clothes I wear?

The above questions illustrate the ways the *intersections* of one's identity are embedded within wider structures that are interpreted by others. These interpretations are outside one's control but usually viewed in isolation to judge someone “what they are perceived to be” rather than being acknowledged for the *interpreted* minor glimpses into one's identity that they are. Taking a holistic approach through intersectionality demands you recognise that there are many “circumstances” in someone's life which are always changing; that is why **we continuously investigate, plan, and reassess.**

**Ethnicity**

**Education**


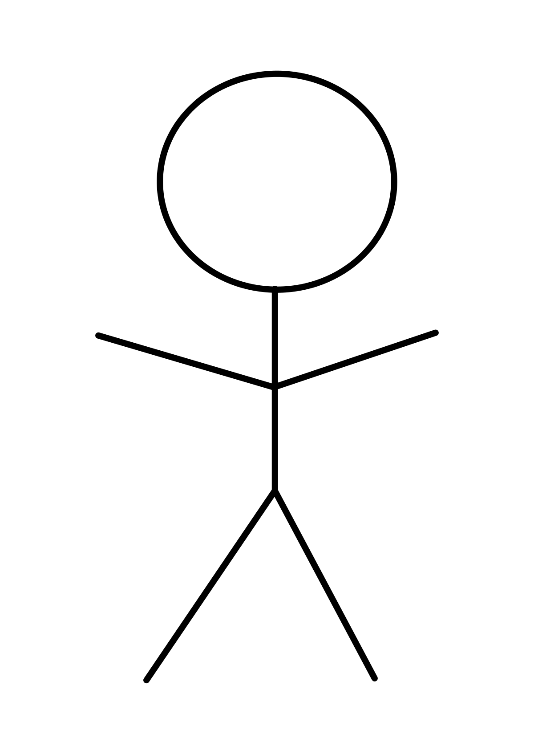


**Neighbourhood**

**Language**

**Class**

**(Dis)ability**

**Occupation**

**Appearance**

**Gender/Sexuality**

**Age**

## The Social Service Team

Recognising the many continuously changing intersections in a child and their family’s life is a great starting point for any holistic practice. Likewise, it takes a **holistic social service team** to respond to the diversity of circumstances, experiences, and expectations of children and families. In our project we will support children and families to access many formal and informal services that we have experience working over the last 10 years.


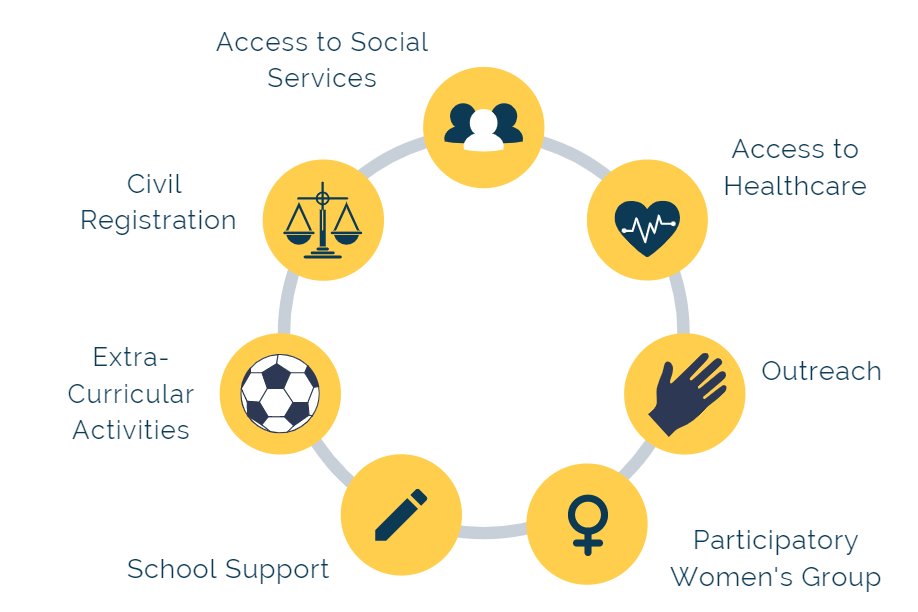


While we discuss each service provided in Guidebook Two, it's important here to recognize and understand the ways the services come together to provide a holistic service to children and families. In our project, our workers will coordinate the delivery the following services:

**Participatory Women's Group** – our women’s project officer will be tasked with coordinating and facilitating the participatory women’s groups across both sites

**Access to Health Care** – medications and RAMED

**Access to Education** – our school support service will liaise with teachers and school directors to ensure children are enrolled in school, and have adequate supplies and clothing to attend school

**Access to Tutoring and Extra-Curricular Activities** – our social integration service connects children and their families with extra-curricular activities and conducts street work activities

**Civil Registration** – all official registration required for children and families is performed through our Civil Registration service

Working within a team is not a straightforward process. It requires constant open and honest communication. There will be a formalised meeting each week to discuss casework and specific service delivery as well as informal sharing sessions between staff to ensure that the team is able to function and effectively deliver services together. Furthermore, the [**professionalism**](#_j1nlwdx2688i) of staff in the ways they work together and perform their roles are facilitated through a clear and extensive case management system which is always up-to-date and monitored. Working together as a social service team is essential for delivering a holistic social service to children and families. Whether it's being prepared for weekly team meetings, returning emails promptly, recording case notes correctly, these are all things which are vitally important to being able to work "**holistically**".

## Collaboration

Collaboration refers to relevant organisations working together to address the needs of the child or young person and their family. Ensuring the safety, wellbeing, and best interests of children and young people is the collective responsibility of parents, families, communities, non-government and government service providers, and legislators.

A range of complex factors characterise many of the families coming into contact with the child protection system, such as low income, unemployment, substance abuse, limited social supports, imprisonment, domestic violence, and mental health issues. Field workers need to connect children and families with services of other government and community-based providers. Accordingly, there may be a need for a number of different services, provided by a range of organisations and workers.

Interagency collaboration in systems of care is the process of agencies and families joining together for the purpose of interdependent problem solving that focuses on improving services to children and families. Delivery of an integrated child protection service relies on individual agencies and professionals working in collaboration with others in the service system, regardless of differences in size, individual philosophies, and structures or funding sources. Adopting a collaborative, integrated approach is fundamental to engagement in a child protection context.

**Effective communication** is a key component for establishing trust. Because collaboration “is not a one-way relationship,” it is important to build trust by recognising both the strengths and limitations of participating agencies. Collaboration requires participants to think bigger and to act beyond their own agency by identifying new ideas and new solutions that are not bound by an individual agency.

# 2. Relational Social Work

Relational social work is about building effective relationships between children and families, and service providers. Relationship building begins with positive engagement and requires clear and effective communication. This is fostered by being empathetic to the circumstances and experiences of all those that you meet. An empathetic approach is fostered through harnessing your active-listening skills and understanding the relationship between trauma, attachment, and transference.

## Engagement

Our approach begins with effective, honest, and positive engagement. Engagement is the process of establishing effective working **relationships** so that there can be a shared understanding of goals and a shared commitment to supporting the child and their family to realise those goals and create sustainable and positive change. **Family engagement through relational social work is the foundation of good child protection practice that promotes the safety, permanency, and wellbeing of children and families in the child welfare system.** Family engagement is the foundation from which change occurs. To build on a family’s resources and kinship connections, family engagement activities focus not only on the immediate family but also on active involvement of both parents, extended family, and the family's natural support systems. Together the 5 Pillars provide a template for you to use to promote and foster effective engagement in all aspects of your work.

**Effective** engagement enables a productive relationship to develop between a worker, the child, and their family, with an aim to:

- provide the family with access to the right combination of services
- enhance social inclusion within the family structure
- ensure the child and the family can exert agency to shape their journey of care
- ensure the family has opportunities to build on their capacity to address issues in relation to providing care and protection to their children
- create a positive, collaborative, effective working relationship between the worker, child, and family to ensure the safety, wellbeing, and best interests of the child
- ensure the family understands and provides feedback on what is happening in circumstances that can often be challenging and distressing

Developing **open**, **honest**, and **positive** relationships between a child, their family and workers is important for:

- providing access to the right combination of services
- productive two-way communication to enhance collaboration with the family in decision making, goal setting, and case planning
- transitional and clear pathways to be known to the family which focus on solutions rather than crisis to avoid dependency
- ensuring the best interests of the child are always upheld
- fostering professionalism and a greater sense of control by the family by maximising accountability and transparency of workers and organisations.

Engagement in a child protection context is based on **best-practice principles** that should underpin the actions to improve the **safety**, **wellbeing**, and **best interests** of a child. All engagement activity is underpinned by our 5 Pillars and should be:

| **Holistic** | in that it accommodates and provides support for a range of different factors |
| --- | --- |
| **Relational** | through clear and appropriate communication to facilitate collaboration between children, families and all service providers |
| **Transitional** | by having a clear way of getting from entry to exit points by adopting a Strengths-Based Solutions Focused approach to avoid dependency |
| **Child-Centred** | by understanding the needs of the child come first and are best met by also adopting a **family-focused** approach |
| **Professional** | by acknowledging our accountability and being transparent with children, families, internal and external service providers, and the wider community |

## Communication: An Empathetic Approach

Effective communication recognises the inherent inequality in the relationship between a child protection worker and a family. Being clear at the start of engagement about the purpose and parameters of a worker’s involvement, their expectations, as well as those of the child or young person and their family, the strategies to be used, and the expected outcomes are essential first steps in good communication.

In addition to communication skills, effective communication with families requires an **empathic approach**. Empathic workers create less resistance and increase the amount of information disclosed by parents involved with the child protection system; it does not mean you do not identify and discuss concerns with parents. Empathy is central to good communication in child protection situations.

The personality and character of workers are more central to good outcomes for children and families than their functionality as “child protection officers”. Even when families may be hostile towards interventions, they can still engage positively with workers when the right qualities are present. Genuineness, empathy, helpfulness, willingness to listen, being non-judgmental, and acceptance are qualities that are cultivated by all workers. These qualities are fostered through harnessing active listening skills and by asking the right open-ended questions.

## Trauma, Attachment and Transference

While we have discussed the importance of identifying and reflecting on the intersection of a person's experiences and identity through a [**holistic approach**](#_xbj2io4nc2sl), here we will discuss the ways past experiences can affect a child, their family, and the care provider. Trauma, Attachment, and Transference are three interrelated concepts regularly used to understand the ways one's past experiences can manifest in the present and influence their future. While we provide a more detailed description of these concepts in the face-to-face training**,** here it’s important to understand the ways the concepts of Trauma, Attachment and Transference manifest in our work.

### Trauma

Trauma in infancy and childhood occurs when a sudden unexpected intense experience overwhelms the coping and defensive mechanisms, creating a feeling of utter helplessness. Early childhood trauma has been associated with reduced size of the brain cortex and may affect IQ and the ability to regulate emotions. The child may become more fearful and may not feel as safe or as protected. Experiencing trauma in childhood can have a severe and long-lasting effect. Children who have been traumatised see the world as a frightening and dangerous place. When childhood trauma is not resolved, the sense of fear and helplessness carries over into adulthood. Young children are particularly vulnerable to witnessing and experiencing violence, abuse, and neglectful circumstances. In chronic and extreme circumstances, children will show a complex trauma response and give meaning to their experiences by believing the abuse was their fault and that they are inherently bad. However, it is not just individual experiences of trauma that influence a child’s ability to understand themselves and others.

### Attachment Theory

Experiences with caregivers have a long-lasting impact on child brain development, their ability to learn, capacity to regulate emotions, and form satisfying relationships. The emotional bonds formed between the child and the parent/caregiver impact the types of attachment developed. A child with **secure attachment** is more likely to be able to develop healthy relationships and a child with **disordered attachment** may find it difficult to sustain healthy relationships even in their adult life. The child’s attachment relationship with their caregiver is what leads to the development of mental representations called the ‘**internal working model**’. This is a cognitive framework for the child to understand themself, other people, and the world about them. The infant will develop the innate ability to expect certain responses from others, according to their past and present experiences with attachment figures. Different internal models of attachment develop in response to the sort of caring the infant and child experiences:

| Attachment | Child’s General State | Parent/Carer Response | Child’s Internal Model |
| --- | --- | --- | --- |
| **Secure** | Secure, explorative, happy | Quick, sensitive, consistent. | Believes and trusts their needs will be met |
| **Ambivalent** | Anxious, insecure, angry | Inconsistent; sensitive vs neglectful | Cannot rely on their needs being met |
| **Avoidant** | Not very explorative, emotionally distant | Distant, disengaged | Subconsciously believes their needs probably won’t be met |
| **Disordered** | Depressed, angry, completely passive, nonresponsive | Erratic, frightening, passive or intrusive | Severely confused, no strategy to have their needs met. |

### Transference

Transference brings together the notions of trauma and attachment to understand the ways current relationships and interactions are shaped through the “transference” of experiences in previous relationships. Such an understanding of relationships enables all staff to be aware of the ways children and families may respond to interventions through previous traumatic experiences.

It can sometimes be difficult to have the same empathy that we have for children towards adults. Adults are not bad people but require you to practice the qualities promoted in a **relational approach**; genuineness, compassion, empathy, willingness to listen, being non-judgmental, and acceptance. Therefore, when working with adults it can be useful to acknowledge the impact of trauma and identify the different attachment patterns to understand the ways adults may also engage in relationships with you and others in ways that are shaped by their previous experiences. For example, a child who experiences neglect and/or abuse from a caregiver may not value and/or feel like they deserve positive relationships as an adult. They may find it difficult to accept a positive attachment and may become deceptive or recalcitrant towards you as a service provider. However, practicing a **relational approach** will help you look beyond and overcome personal bereavements and project the empathy and understanding required to connect with and support them develop positive attachments with you, others, and most importantly themselves.

Transference is not just a way to understand experiences of children and their family. It is vitally important for workers to be able to **reflect and recognise** the transference that can occur when providing services to children and their families in difficult situations. Regular supervision with a dedicated manager as well as working as a [**team**](#_54o11mvkxsgd) to plan interventions are steps taken to limit the types of transference which can impede a service provider's judgment. Lastly, we have available a dedicated psychologist as part of our service team who is able to support staff through second-hand trauma that they may experience when conducting casework and who is able to provide their expertise on cases of trauma and attachment within casework.

# 3. Transitional

## Entry and Exit Points

**Promoting a transitional relationship directly challenges the dependency on services common in any social welfare system.** It is important to remember ways of identifying children and families who are within our entry points and when they are no longer or are no longer at risk of falling in our entry points. Our entry points are directly related to the [**holistic service**](#_xvpa0ixveg6b)  we provide and are:

1. Difficulty attending or enrolling in School
2. Do not have access to RAMED
3. Do not have access to Civil Registration

The **flow chart** on the next page provides a map of the ways children and families will be identified as meeting our entry points and supported to travel through our services to reach our exit points. This process draws on our 5 Pillars to ensure we are:

| **Holistic** | in the ways we are acknowledging the embeddedness of children and families in wider social environment |
| --- | --- |
| **Relational** | being aware of the interrelation of the past, present, and future relationships of children and families |
| **Transitional** | providing a clear structure for assessment and continuous reassessment |
| **Child-Centred** | placing the rights and interests of children at the forefront of any intervention |
| **Professional** | following best practice and are evidence-based by ensuring our assessment process and ways of working are only diverted from through approval from appropriate team members. |

See the flow chart on the next page for a detailed understanding of the transitional journey children and their families embark upon from our entry to our exit points.


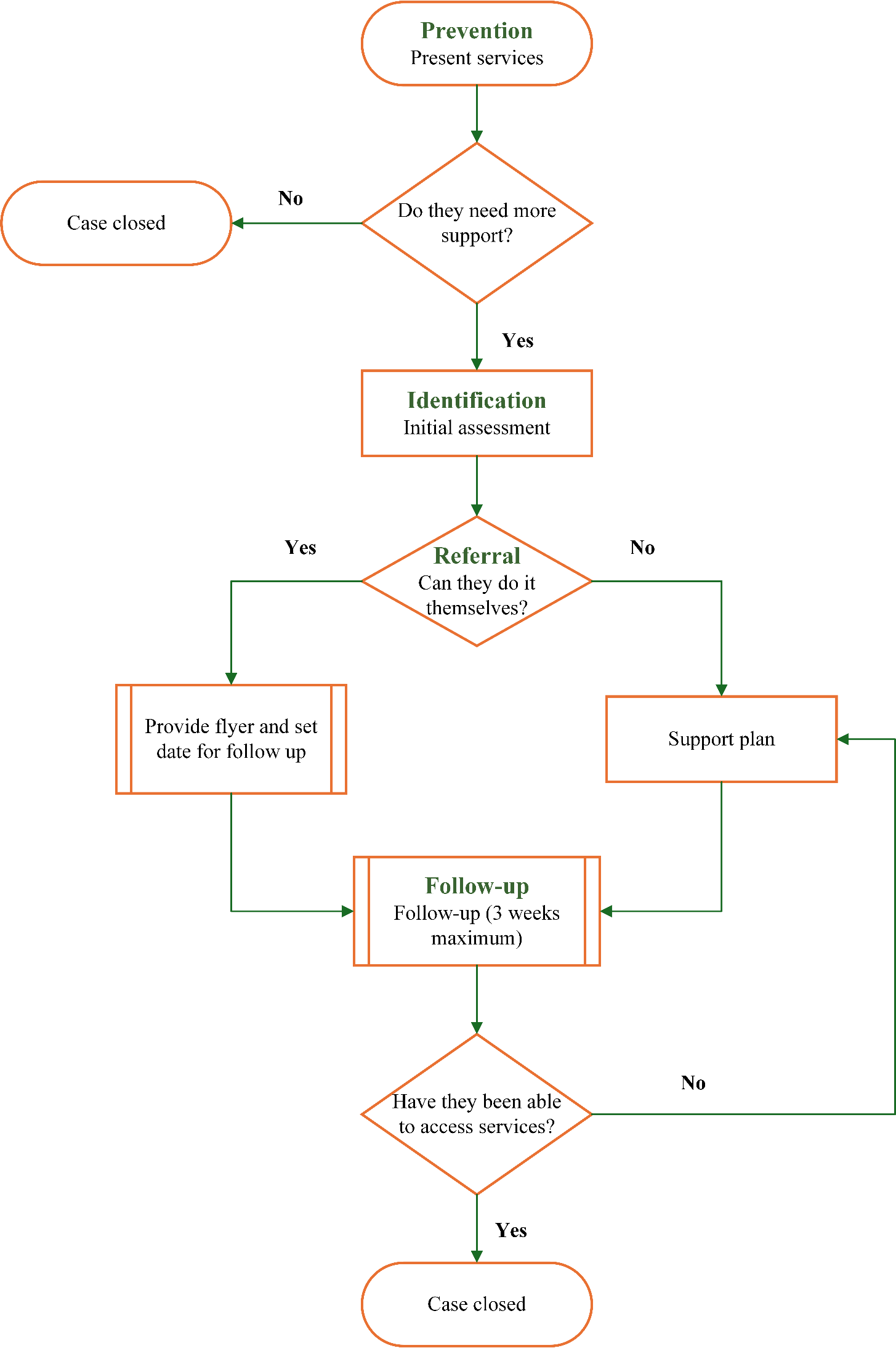


## Participatory

To avoid dependency on services it is important to ensure children and families relationship with service providers is participatory and transitional. Participation is an essential aspect of the transitional journey taken through all child protection services. True participation for families includes the right to information and knowledge held by professionals, the opportunity to identify solutions within a supportive familial and community context, and the right to self-agency supported by respectful professional services. Importantly, it also involves **providing the child and their family with the confidence and skills to effectively participate**. This means you must be able to reflect on the position of power (strengths!) you hold as care providers and always actively seek ways to include and value the voices of all family members in the decision-making process. We will cover the ways we actively seek the participation of children and their families in the monitoring and evaluating of our services in **Guidebook Two.**

## Strengths-Based Solutions Focused

A Strengths-Based Solutions Focused (SBSF) approach operates on the assumption that all people, even if they are experiencing problems, have some strengths and resources from which they can draw on to make positive change. A deficit-based approach, which focuses on what is wrong, can overlook valuable skills and experiences a family has. It can also reduce a family’s motivation to actively engage with services and impair the likelihood of positive change for children.

### What does strengths-based mean?

When adopting a strengths-based approach the following points may provide assistance:

- try to identify what a child or family is doing well or what personal resources they may have as these strengths could influence the identification of appropriate services or supports
- ask the child or family what they believe their strengths are
- use positive language as the right words and language can be empowering
- use strength-based skills, such as:
  - allowing the child or their family to tell their story without interruption
  - highlighting strengths
  - exploring and addressing structural and personal constraints
  - identifying significant people who can support the child and/or family
  - developing a picture of the future and establishing realistic goals
  - actively identifying and measuring change and progress
  - identifying achievable steps and strategies
  - celebrating successes

When engagement uses a strengths-based approach, the following indicators of success may be present:

- communication is open and information is being exchanged
- appointments are kept both by the worker and by the family
- ideas are generated on ways to address issues or possible programs and activities that could help
- the child and/or their family listens, considers suggestions, and may make their own suggestions
- the child and/or their family takes responsibility for following up on agreed actions
- the family is keeping the worker updated on their progress and advises of changes in circumstances including contact details and personal information

A simple understanding of how a **Strengths-Based Solutions-Focused** approach is used in practice is to remember the following three questions in your everyday work when creating plans, deciding on interventions, or when problem solving with other service providers both internally and externally:

1. **What worked before?**
2. **How did it work?**
3. **How can we apply the knowledge learned to the present?**

The logic of the above three questions can be repurposed to navigate unforeseen situations and circumstances field workers and managers experience.

### Circles of Support

You should always follow a clear logical **SBSF Child-Centred Family-Focused** approach when conducting an assessment or performing any intervention. This is to **avoid dependency** on services, setting impossible goals that family members do not have the capacity to reach, and to ensure you are always drawing on the **family’s own resources** – i.e. strengths! – to drive the type and level support required. The **Circles of Support** are a way to ensure you are following a clear logical progression of thinking when coming to decisions at each stage of your relationship with each member of the family. The Circles of Support prioritise:

1. The **individuals** own strengths, desires, and capacities at various levels.
2. The “strengths” found within the **family** – i.e. this entails all members of the family being aware of each-other’s strengths, desires, and capacities at various levels.
3. The family’s wider **supporting network** – e.g. includes extended family, friends, or neighbours.
4. **New formal official partnerships** we are to support them making with (non)government service providers.

Figure 1. Circles of Support

**New formal/official partnerships**

**The family’s wider support network**

**The family group**

**Individual family member**

# 4. Child-Centred

## Being Child-Centred and Family-Focused

Adopting a child-centred and family focused approach is not only important in terms of enhancing the realisation of positive outcomes but will improve the likelihood of effectively engaging children and their families. **Child-centred and family-focused approaches are not mutually exclusive.** While ‘child-centred’ refers to placing the needs of the child at the heart of any decision, being ‘family-focused’ recognises that the issues and needs of parents will impact on the child.

### Child-Centred

Adopting a **child-centred** practice means:

- Linking children, particularly very young children and their families with services and supports will strengthen their physical, cognitive, and social functioning.
- Thorough assessments of children and young people need to take account of their developmental level across a range of spectrums.
- All interventions should seek to create and strengthen children’s networks and include the provision of appropriate information to enable the child’s networks to increase protection and support.
- Interventions involving young people should recognise the importance of the young person developing a sense of self and that it may not always be appropriate for the family to be involved in the resolution of adolescent issues.

### Family Focused

Part of being [**Holistic**](#_xvpa0ixveg6b) is being family focused. Adopting a **family focused** practice means:

- being inclusive and involving parents, extended family, kin, and friends (where appropriate and relevant) as well as recognising and considering the role of the broader community
- understanding the child’s position in the family in relation to other family members
- ensuring where possible that there is two-way communication between the family and worker, especially regarding decision-making
- developing a knowledge and understanding of the family's past experiences, current situation, concerns, and strengths to inform case plans based on an assessment of the child and parent’s strengths and needs
- that case plans reflect ongoing input from the family and are specific, measurable, achievable, realistic, and timely
- understanding that the combination of institutional mistrust with the complex issues that families face, for example, substance use, health issues and violence, is a serious impediment to the engagement families with services
- recognising that engaging fathers in the child protection context may require different considerations and approaches to those adopted when working with mothers
- providing or facilitating the provision of concrete services to meet immediate needs for food, housing, child-care, transportation, and other costs, and help communicate to families a sincere desire to *support* rather than *help*.

# 5. Professional

Promoting and practicing professionalism in all aspects of our work ensures our rights and responsibilities as service providers are upheld. We promote and practice professionalism through our **assessment process**, when maintaining and enforcing **procedures and policies** regarding child safe ways of working, by ensuring **caseloads** are fair and manageable for staff, and by providing ongoing **training**, **support**, and **supervision** for all staff and management.

## Vision and Purpose

It's important to be able to understand and be guided by a common vision and purpose. We draw on Moroccan and international expertise in public health, social work, paediatrics, education, safeguarding, and children's rights to fulfill our major objective which is to **promote and provide social support for children and families**. Our work hopes to contribute to the **development of a robust and strong child protection system in Morocco.** Our vision is for an environment where children are protected against all forms of abuse and is hinged on 5 objectives that contribute to the creation of:

1. A Child Protection System in Morocco
2. Advocating for and working towards the structural and everyday processes required for Deinstitutionalization.
3. Promoting and Providing Foster Care
4. Ensuring Civil Registration remains a general right of every Moroccan Citizen.
5. Provide the template for a Children Protection Centre through Centre Amane

## Confidentiality

In our organization, confidentiality is of paramount importance to children and family as well as to employees. We understand that breach of confidentiality can affect the services we provide because our children or a family will be less likely to provide the information we need to help and support them. We want families and children in contact with our organization to be sure that the personal and sensitive information they have confided about their life or family situation will not be shared or passed on to other individuals or organizations without their consent. We maintain a strict ”**no face**” policy for photos of children in our care for all publicity material. We also recognizes that all personal information shared by employees must be respected and treated confidentially so that they can maintain their trust in the organization.

We are committed to the following principals regarding confidentiality:

1. In the training and supervision we provide to staff
2. Building trust by not sharing information
3. Ensuring all staff read and follow our confidentiality policy
4. Securely collecting and storing information
5. Having a formal complaints procedure

**For further discussion see our confidentiality policy**

## Accountability and Transparency

**Accountability** is the way everyone employed by us has a duty of care to fulfil their rights and responsibilities as care providers relative to their respective positions. Such accountability goes beyond notions of seniority, hierarchy, or differing modes of practice, and rather perceives every individual care provider as part of a **collaborative service team**. However, accountability begins with **transparency**, and we are committed to transparency and honesty about who we are and what we do. We believe a more open, more transparent, more accountable world is the foundation on which we can help contribute to building a more just world for children and future generations.

Together, accountability and transparency refer to the continual assessment of practice, organisational, and financial outcomes to determine the **effectiveness of our systems of care in meeting the needs of children and families.** Fundamentally, this refers to the clear and accurate recording, management, evaluation, and dissemination of information. There are many ways we practice accountability and transparency in our everyday work in fulfillment of our role as child protection actors. The **essential components of our system of accountability and transparency** are:

- A **comprehensive management information system** that tracks important indicators of individual case information, effectiveness of services for those cases, and the ways in which the service team works within those systems. This includes the timely and accurate recording of case work and work schedules in order for fellow service team members to be **fully aware** and **capable** to fulfill their roles.
- An **evaluation strategy** which draws on the ideas of [**participation**](#_3rdcrjn), [**communication**](#_26in1rg), and [**collaboration**](#_lnxbz9) to work with children and families and staff to continuously improve our service delivery and modes of practice internally as well as provide our children and families and the wider community we serve with up-to-date, accurate, and understandable information about our services.
- An **external audit** conducted each year to ensure we align with Moroccan law regarding accounting, human resources, data protection, and in the ways we perform our everyday work.
- A HR Guidebook provided to each employee detailing our policies regarding ways of working, pay scales, leaves and absences, and the rights and responsibilities of employees and management.

**For further information regarding our HR policies see the HR Guidebook**
