# Supplementary material for: Piloting a NGO-led signposting intervention to improve access to government welfare in Southern Morocco: a feasibility study
Source: Int J Equity Health. 2025 Oct 16;24:280. doi: 10.1186/s12939-025-02605-0 (PMC12532411; doi:10.1186/s12939-025-02605-0)
Supplement: Supplementary file 3 — Additional file 3. This guidebook details our referral process and the tools used for recording, assessing, and following up on referrals, including the Work Journal, Initial Assessment, Referral Tracking Document, and Case Journal [file 12939_2025_2605_MOESM3_ESM.docx]

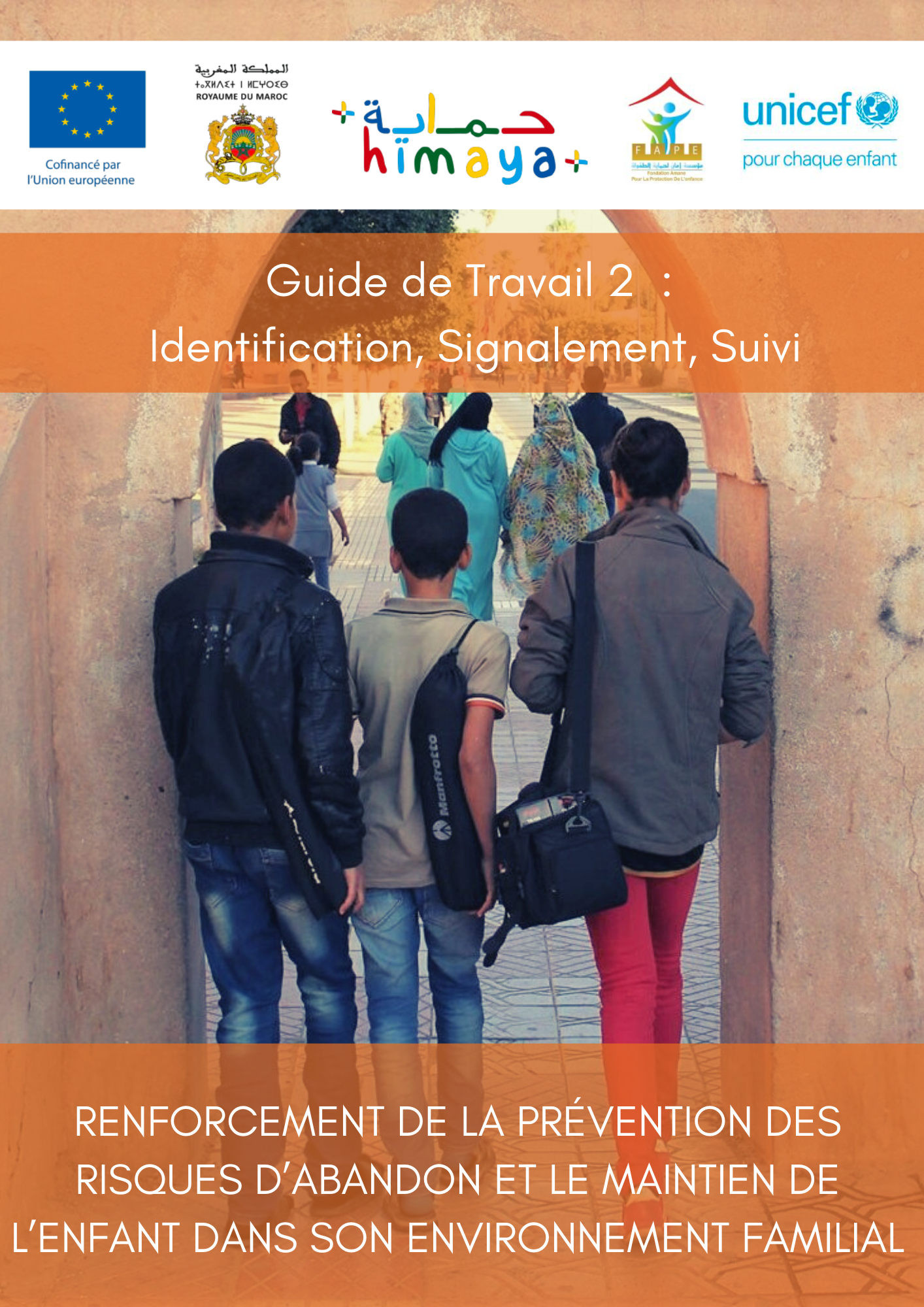


# Table of Contents

[Using this Guide 1](#_Toc97917881)

[Supporting Access to Services 2](#_Toc97917882)

[Civil Registration 2](#_Toc97917883)

[Prevention, Identification, Referral, Follow-Up 2](#_Toc97917884)

[The Process 2](#_Toc97917885)

[Limits: Our Entry/Exit Points 3](#_Toc97917886)

[Rights and Responsibilities as Child Protection Actors 3](#_Toc97917887)

[Recording your work 6](#_Toc97917888)

[Working in a Team 6](#_Toc97917889)

[Work Journal 6](#_Toc97917890)

[Organising and Planning Activities 7](#_Toc97917891)

[Initial Assessment 7](#_Toc97917892)

[Number of Referral 7](#_Toc97917893)

[Site 7](#_Toc97917894)

[Personal Details 7](#_Toc97917895)

[Children 7](#_Toc97917896)

[Service required 8](#_Toc97917897)

[Referred to 8](#_Toc97917898)

[Initial Assessment Tracking 8](#_Toc97917899)

[Column A-W 8](#_Toc97917900)

[Column X-AA 8](#_Toc97917901)

[Case Journal 8](#_Toc97917902)

## Using this Guide

In this guide we will discuss our referral process and the tools we use to record, assess, and follow-up referrals. These tools consist of using the **Work Journal**, completing the **Initial Assessment,** completing **The Referral Tracking Document, and** the **case journal**. Therefore, it is strongly recommended you have all tools on-hand while reading through this guide. Significantly, the referral process does not end with your final decision or recommendation. **Following up** any referrals is vitally important and the ways we conduct our follow up will also be discussed.

Our identification and referral processes are the cornerstone of our project. This process begins by ensuring we have a presence at key intake sites in each location to ensure people who require our support be able to access it. These sites consist of **schools**, **civil society associations**, and **public spaces** in the neighbourhoods of **Drargra** and **Lqila**.

**IMPORTANT**: it is important here to remember why and the ways referrals are signalled to our project and the **limits** of our role. A good way to ensure we are staying within our limits is to remember the **major objective** of our project – to **support children and families to gain access to services**. Detecting and working with children in difficult circumstances can be difficult due to:

1. Lay community members not being trained in child protection and therefore may not have the tools or resources to be able identify and report abuse or when children are at risk of abuse
2. Abuse may occur within families or closely knit communities making whistleblowing extremely rare and sensitive
3. The legal processes to receive and process referrals as a service provider are not straight forward and those able to provide authority for an association to work with children and families are exclusively the prosecutor, police judiciary, or local authorities. Even if you witness signs of abuse, you must approach and obtain official approval from the designated authorities to have the right to work with children and families

# Supporting Access to Services

In our project we will be supporting children and families to access key services. These services are civil registration, education, and health. Together, access to these services support in allowing children and families to access the full potential of their citizenship as Moroccans, reduces the street connection of children, and reduces the risk of abandonment and institutionalisation.

## Civil Registration

The lack of an official identity card – Civil Registration – affects almost every aspect of the lives of unregistered Moroccan children. It condemns them to a lifetime of secondary citizenship, unable to access social services entitled to them by birth, such as education, social and health services, official employment, and the ability to travel overseas. Due to the close partnerships with local stakeholders and the advocacy awareness workshops from the previous CARE project, we have been able to develop and hone our ability to assist children and families who are unable to access Civil Registration. The Civil Registration service is fundamental to our **holistic** and **transitional** service as it allows children and families to gain access to social entitlements and future opportunities helping to break the cycle of poverty. It means children can be enrolled in school and gain **TAYSIR**, access government subsidised health care through **RAMED**, and enter employment when they come of age. Therefore, in our project we will begin with ensuring children and families are able to access Civil Registration, before ensuring they can access the other social security provided by the government; **TAYSIR** and **RAMED**. The procedures to complete the registration for the different types of social security will be shared in the training which accompanies this guidebook.

# Prevention, Identification, Referral, Follow-Up

## The Process

Our project will follow a clear and logical progression of working with children and their families. See the flowchart on page 4 where we detail the four stages of our process, the questions to ask at each stage, and the recording tools used at each stage which are:

**Stage 1**: **Prevention**: where we conduct awareness raising activities at our sites and provide key information leaflets and instructions on how to access services.

**Stage 2**: **Identification**: conduct an initial assessment where we identify the types of support required by children and families

**Stage 3**: **Referral**: ensure children and families can access support

**Stage 4**: **Follow-up**: ensure children and families have been able to receive the support they required.

## Limits: Our Entry/Exit Points

It’s always important to be aware of our entry and exit points to ensure we can work within our limits and provide the best services to children and families within our capacities. We have provided a list of questions and answers that you should be constantly referring to when working with children and families in the project. See table on page 4.

## Rights and Responsibilities as Child Protection Actors

If at any stage, you **become aware of or suspect a child is in danger it is your responsibility to report it** to your supervisor immediately who will support you in following the necessary steps. Different child protection issues require different services, different procedures, and different levels and types of follow-ups. The important thing is to **inform your supervisor** of any child protection issues **before** advising the child and/or family of any commitments we can make and before contacting any external child protection actor to inform them about the case.


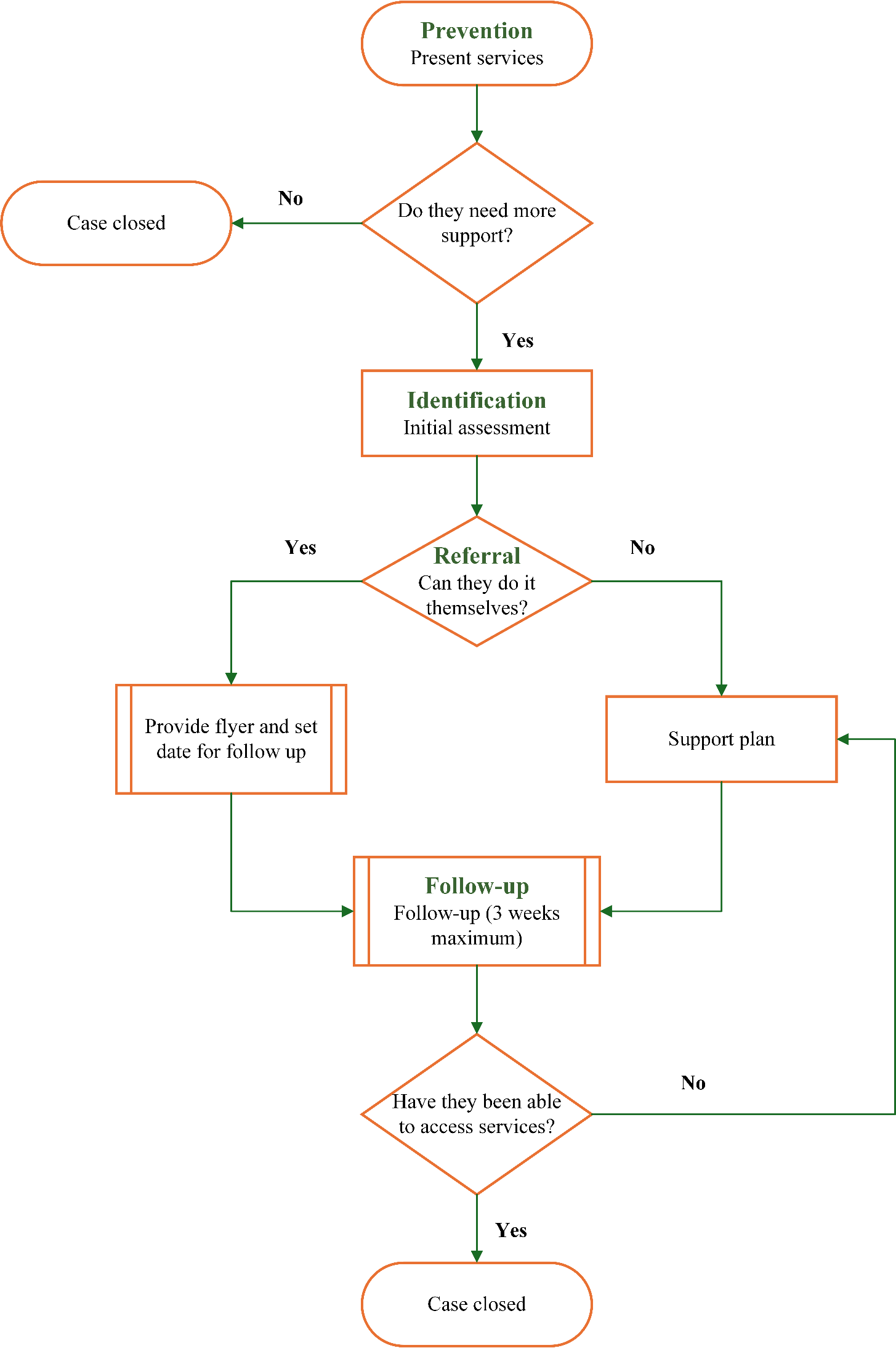


| **Question** | **Civil Registration** | **Education** | **Health** |
| --- | --- | --- | --- |
| **When do we enter?** | When they do not have civil registration | - When children are not enrolled or dropped-out of school - When children do not have access to TAYSIR | When they do not have RAMED |
| **When do we NOT enter?** | We will support all civil registration cases within our capacity | When children are suspended or expelled, having trouble with peers and/or teachers, having difficulty with their grades | We will support all RAMED cases within our capacity |
| **How do we work when we enter?** | We provide advice and guidance (such as the documents required) to follow the procedures to obtain civil registration | - We provide advice and guidance – such as the documents required for Taysir or who to contact for enrolment – to follow the procedures to enrol children into school and will be able to contact teacher/school-directors on their behalf. - We will provide bags, school clothes, and school equipment for select children which you must gain approval from your supervisor before providing. - If required, we will connect children with local associations providing tutoring and extra-curricular activities. | - We provide advice and guidance (such as the documents required) to follow the procedures to obtain RAMED. - We will also provide funds for medical procedures or pharmaceuticals for select families which you must gain approval from your supervisor before providing. |
| **How do we NOT work?** | We don’t conduct visits to villages/ homes/authorities to collect paperwork on behalf of cases | We don’t conduct visits to villages/homes/ authorities to collect paperwork on behalf of cases or provide tutoring services to children | We don’t conduct visits to villages /homes/authorities to collect paperwork on behalf of cases |
| **When do we close?** | When we have been able to support the person to obtain civil registration or referred them to another service provider | When the child is enrolled in school | When we have been able to support the person to obtain RAMED or referred them to another service provider |

# Recording your work

## Working in a Team

Working within a team is not a straightforward process. It requires constant open and honest communication. The team consists of:

**Project Manager:** coordinating the project activities and team, ensuring progress against the project objectives (by following the performance indicators), developing the action plans, identifying corrective measures when necessary, drafting of reports, and ensuring adherence to the budget.

**Administration and Finance Officer**: responsible for all the project accounting and Human Resources

**Field Officers**: field officers are responsible for all the frontline work including presentations, processing, and following up referrals.

All team members will meet regularly with the coordinator via google meets. However, all staff will come to Taroudant periodically for further training and larger team meetings. These meetings are a place for you to share your successes and work together to overcome any challenges that you may be facing. However, it’s not just in formal weekly team meetings that will require you to work together, constant open and honest communication amongst all field officers and project management ensures you can be supported when it’s required and, in many instances, before you may even think it is required.

## Work Journal

The work journal is an important tool to plan, manage, and record the logistics of your everyday work. It is not just your supervisor who will monitor your journal but having an up-to-date and accurate journal enables other team members to plan their work and any potential collaboration.

Everyday meetings, phone calls, and interactions with other child protection actors are vital to the informal relationship building required for a functioning and robust child protection system. It enables a link to be created between actors across services and organisation in order for children to be referred, tracked, and supported through an array of services. Therefore, the work journal contains dropdown boxes for each day which tally up the number of face-to-face meetings conducted with other child protection actors – e.g., teachers, doctors, social workers, health workers, authorities.

## Organising and Planning Activities

Organising activities is not a straightforward process. There are many processes and steps required to ensure you are prepared and everything is organised. We have provided you with a checklist you can use to ensure you are prepared **here**. There is also a tracking document to log the preparation and completion of activities that you can find **here**. It is the responsibility of both team members, whether you are designated as the facilitator or notetaker, to ensure the steps required for preparations of the workshops are followed.

## Initial Assessment

### Number of Referral

To begin, in the top right of the document please be sure to clearly number each initial evaluation by following the progressive numbering system found in the Referral Tracking Document. Begin with the unique number, followed by a solidus (/) and the year – e.g., 50/2020. Having a clear and accurate numbering system will help ensure referrals can be identified in the future.

### Site

Next you must write the site in full. If unsure of the complete site name check the tab ‘overview’ in the referral tracking document.

### Personal Details

Please be sure to complete all personal details as you will need to be able to report the gender and ages of all participants and you may need their number in the future to conduct a follow-up call.

### Children

This table asks you to list all the children in the family. Please provide full names, ages, and gender. Then you must tick the box that corresponds with the service they **do** have.

### Service required

This is **not** a place to tick or simply write yes or no, but you must explain the details of why they need the service and what the hurdles may be.

### Referred to

This is **not** a place to tick or simply write yes or no, but you must explain who you have referred them to, provide their official title/organisation, name, and contact number. If they ‘can do it themselves’ next to self-service write how and if we will be working with them, next write how and the date agreed to conduct a follow-up. Remember the follow-up date cannot be greater than 3 months.

## Initial Assessment Tracking

### Column A-W

Columns A-W require you to simply type the information you have written down in the Initial Assessment. Several of the columns contain drop down boxes to save you time in recording and ensure there is uniformity in recording to allow for accurate tracking over the year. Columns I-R just ask you to record the number of children or adults that correspond with the age and gender in the family; do not include the main contact have entered in Column e in this count.

### Column X-AA

The follow-up date you place in column X cannot be greater than 3 weeks from the date of referral. However, you must follow up all referrals regardless of the support provided and write the outcome, and close date. If a case is integrated more fully into our project, please request a case file and link the case file to this document.

## Case Journal

Together with the **Work Journal**, the Case Journal supports you to plan, organise, and keep track of the work you need to complete and the work you have completed. Each family has its own Case Journal. There is a landing page which provides basic information such as address, phone number, ages, genders, and their access to services.

The tab “**Family Plan**” is where the team will log the plans as they develop for each case. The “**Status**” column allows you to track the progression of each intervention; urgent, opened, complete, in need of redevelopment, or in-progress. The dependent drop-down lists in column J & K were created through extensive work with frontline workers and management to determine the main areas of work. Significantly, the final column here allows the team to dictate who is tasked with the intervention.

The tab “**Interventions”** is where you can record the actions the day-to-day case work. It shares a similar structure to the previous tab, though contains an additional column “**Where**” that records where/how each intervention takes place. Importantly, there is space for you to indicate what the next steps are.

**Remember:** when negotiating new plans with your team in the “Family Plan” tab, it will not be enough to just speak about the actions you have completed, **you need evidence of these actions**. The way you provide this evidence is to ensure that you record these actions in detail in “Interventions”. Accurate, clear, and timely recording of actions will also provide you with a useful reservoir of information when making plans, conducting reviews, or re-familiarising yourself with the history of a case.

The tab “**totals**” records the monthly totals of each type of intervention and significant information such as listening session and home visits can be monitored for each family.

A way of understanding the relationship between the different journals and tabs is:

| **1** | “**Plan**” – **Cas Journal**  Where you and the team will document in team meetings **what** actions need to occur and **why** you will perform each action |
| --- | --- |
| **2** | **Work Journal**  Where you plan and record **how** you perform(ed) the action – i.e., the logistics such as time management and transport |
| **3** | “**Interventions**” – **Case Journal**  Where you document the **performing** and **outcome** of the action |
